# Exploring the Potential Health Impact and Cost-Effectiveness of AIDS Vaccine within a Comprehensive HIV/AIDS Response in Low- and Middle-Income Countries

**DOI:** 10.1371/journal.pone.0146387

**Published:** 2016-01-05

**Authors:** Thomas M. Harmon, Kevin A. Fisher, Margaret G. McGlynn, John Stover, Mitchell J. Warren, Yu Teng, Arne Näveke

**Affiliations:** 1 International AIDS Vaccine Initiative, New York, NY, United States of America; 2 AVAC, New York, NY, United States of America; 3 Avenir Health, Glastonbury, CT, United States of America; Commissariat a l'Energie Atomique(cea), FRANCE

## Abstract

**Background:**

The Investment Framework Enhanced (IFE) proposed in 2013 by the Joint United Nations Programme on HIV/AIDS (UNAIDS) explored how maximizing existing interventions and adding emerging prevention options, including a vaccine, could further reduce new HIV infections and AIDS-related deaths in low- and middle-income countries (LMICs). This article describes additional modeling which looks more closely at the potential health impact and cost-effectiveness of AIDS vaccination in LMICs as part of UNAIDS IFE.

**Methods:**

An epidemiological model was used to explore the potential impact of AIDS vaccination in LMICs in combination with other interventions through 2070. Assumptions were based on perspectives from research, vaccination and public health experts, as well as observations from other HIV/AIDS interventions and vaccination programs. Sensitivity analyses varied vaccine efficacy, duration of protection, coverage, and cost.

**Results:**

If UNAIDS IFE goals were fully achieved, new annual HIV infections in LMICs would decline from 2.0 million in 2014 to 550,000 in 2070. A 70% efficacious vaccine introduced in 2027 with three doses, strong uptake and five years of protection would reduce annual new infections by 44% over the first decade, by 65% the first 25 years and by 78% to 122,000 in 2070. Vaccine impact would be much greater if the assumptions in UNAIDS IFE were not fully achieved. An AIDS vaccine would be cost-effective within a wide range of scenarios.

**Interpretation:**

Even a modestly effective vaccine could contribute strongly to a sustainable response to HIV/AIDS and be cost-effective, even with optimistic assumptions about other interventions. Higher efficacy would provide even greater impact and cost-effectiveness, and would support broader access. Vaccine efficacy and cost per regimen are critical in achieving cost-effectiveness, with cost per regimen being particularly critical in low-income countries and at lower efficacy levels.

## Introduction

Since its discovery in the early 1980s, HIV has infected almost 80 million people across the world and 40 million have died from AIDS-related causes. Strong international commitments, generous funding, remarkable advances in treatment and prevention, and broad social mobilization have bent the epidemic’s trajectory, with 2014 showing a 42% decrease in annual AIDS-related deaths since their peak in 2004, and a 35% decrease in new infections since 2000. However, in 2014 2 million people were newly infected with HIV, with 36.9 million people living with HIV overall. 1.2 million died of AIDS-related causes in 2014 [1]. Low- and middle-income countries (LMICs) are the most severely affected, especially in Sub-Saharan Africa, where 70% of new HIV infections and AIDS-related deaths occurred in 2014 [1]. Prevalence in the general adult population can be as high as South Africa’s 18% and Swaziland’s 27% [2]. Incidence in young women and girls in sub-Saharan Africa is twice that of boys and men their age [1]. In other countries with lower rates in the overall population, prevalence among commercial sex workers, men who have sex with men, transgender people and injecting drug users can be 12–19 times higher than that of the general global population [1, 3].

Antiretroviral treatment (ART) has saved millions of lives from AIDS and can also help reduce new infections by decreasing the amount of virus circulating in the body [4]. However, ensuring ART access and adherence represents a logistical, financial and behavioral challenge, with even high-income countries seeing a wide disparity between the number of people living with HIV and those knowing their status, accessing and adhering to treatment to the point of viral suppression [5]. Recent updated guidance by the World Health Organization (WHO) recommends initiation of ART at the time of a positive HIV diagnosis, and 15.8 million people living with HIV (less than half of the total) accessing treatment by June of 2015 [6, 7]. With 2 million new infections a year contributing to the mounting number of people living with HIV, accelerating necessary progress will require enhanced roll-out of available prevention options, such as such as male and female condoms, oral pre-exposure prophylaxis (PrEP), and voluntary medical male circumcision, and of new options, including other forms of PrEP, treatment as prevention (TasP), microbicides, and a vaccine.

Developing an AIDS vaccine is a huge scientific challenge. HIV mutates very rapidly, allowing the virus to escape the body’s immune responses while giving rise to multiple clades that circulate around the globe. Further, HIV establishes persistent infection quickly following transmission, suggesting that the opportunity to prevent or abort an infection is short-lived. Of the few vaccine candidates that have progressed to late-stage clinical trials most have failed to demonstrate efficacy [8, 9]. In 2009, combining the ALVAC and AIDSVAX candidates reduced new infections by 31% after three years of follow-up in the Phase III RV144 trial in Thailand [10]. An efficacy study of a redesigned version of this candidate is planned to start in late 2016 in South Africa, including modification to clade C, a new protein boost, different adjuvants, and additional booster shots intended to improve both strength and durability of protection [11]. In addition, there are currently more than 30 earlier-stage clinical studies underway around the world to investigate other vaccine candidates aiming at eliciting strong, broad, and lasting efficacy [12].

Models combining epidemiological information, effectiveness of existing and new interventions, and cost help guide rational, strategic decision-making on the expansion of HIV/AIDS programs. In 2011, the UNAIDS Investment Framework (IF) was published, providing guidance to donors and LMICs of varying epidemic types toward enhancing existing prevention and treatment options to further reduce new HIV infections and AIDS-related deaths by 2015 [13]. UNAIDS’ Investment Framework Enhanced (IFE; developed between 2012–13) included further scale-up of ART according to revised WHO guidelines and explored potential contributions of emerging prevention technologies (NPTs), including PrEP, TasP, and a potential AIDS vaccine [14]. In 2014, UNAIDS’ “90-90-90” campaign proposed that by 2020 90% of all people living with HIV know their HIV status, 90% of those HIV-positive people who know their status receive ART, and 90% of all people receiving ART have achieved and sustained viral suppression [7].

This modeling study builds on UNAIDS’ IFE, as detailed in Stover et al. 2014, to provide a more detailed analysis of the potential impact and cost-effectiveness of a vaccine in reducing new HIV infections and AIDS-related deaths in order to help inform decision-making by donors, policymakers and researchers toward investment and critical success factors in AIDS vaccine research and development.

## Methods

### Vaccine model

This project utilizes the same Goals model (part of the Spectrum software package) and core primary assumptions as the UNAIDS IFE [13, 14]. Goals models the potential impact of different interventions in 24 countries accounting for 85% of new HIV infections in LMICs based on UNAIDS estimates. Goals is a compartment model that categorizes sub-populations by behaviors, in this case sexual contact and needle-sharing, and simulates the transmission of HIV. Its structure is similar to other compartment models (although the specific population groups included differ across models) and it differs from microsimulation models that create discrete populations of individuals and characterize each individual with randomly assigned characteristics based on population data [15]. The model is fit to the historical epidemic from 1970 to 2013. A previously developed vaccine module was utilized to conduct vaccine-specific follow-on modeling to further explore the potential impact and cost-effectiveness of a vaccine in reducing new HIV infections and AIDS-related deaths in LMICs, sensitivity to basic product and implementation characteristics, corresponding impact on ART needs and total costs of the HIV/AIDS response [16]. A more detailed description of the Goals model as utilized in the UNAIDS IFE can be found in the supporting information for Stover et al. 2014 [14].

For generalized epidemics we assumed a temporary catch-up vaccination of older age groups in addition to vaccination of those at or above the target age for routine vaccination. Temporary catch-up programs have been a common characteristic of many vaccination programs to accelerate health benefits. However, the benefits from this investment will take years to fully materialize. The model projects out to 2070, in order to adequately capture the health and cost implications of an AIDS vaccine over time. As a comparison, modeling of the health impact and cost-effectiveness of human papillomavirus (HPV) vaccines has projected out to a period of 100 years [17].

The Goals model focuses on individuals of reproductive age (15–49), and includes new infections occurring due to mother-to-child transmission, but does not yet allow exploration of interventions for individuals younger than 15. Thus, these analyses do not include sexual transmission that may take place before the age of 15. The introduction of vaccination programs in individuals younger than 15 (starting at age 10) required a “delayed” entry into the model of vaccinated individuals as they reached that age. Further, the model did not utilize purely differentiated age tiers as stated in the assumptions, so a calculation of the total average population coverage was used in the model to approximate these tiers. While future iterations of the model will allow for such tiering by age to provide a more granular representation of vaccination programs, this limitation does not overly distort the model’s results.

### Background scenarios

The UNAIDS IFE represents the most comprehensive global HIV/AIDS investment framework published in peer-reviewed literature, and thus was used as the primary fundamental framework for this vaccine modeling. Three iterations derived from UNAIDS IFE were explored as background scenarios:

- A *Current Trends* scenario assumes that incremental linear scale-up of ART and prevention of mother-to-child transmission (PMTCT) from 2010–2013 continues linearly into the future, offsetting the natural increase in new infections associated with population growth in LMICs after 2015 and resulting in a steady annual rate of new HIV infections. Future coverage is capped at 80% for ART and 95% for PMTCT. Eligibility for ART remains at CD4 counts <350 cells/mm^3^, so most countries reach 80% coverage quickly but do not progress to much higher numbers on ART as seen in the IFE, which utilizes more aggressive treatment scale-up thresholds recommended by the WHO in 2012, but which have yet to be implemented in many LMICs [18].
- *50% Scale-up of IFE* assumes that UNAIDS IFE targets are only achieved halfway. The *50% Scale-up of IFE* scenario is based on linear scale up from 2013 coverage to the 50% of IFE target levels in 2020.
- The *Full Scale-up of IFE* scenario assumes that UNAIDS IFE targets are fully achieved, and is based on linear scale up from 2013 to the target levels in 2020. Detailed assumptions for the IFE, including scale-up targets for specific interventions, can be found in Stover et al. 2014 [14].

### Assumptions of vaccine and vaccination characteristics, and sensitivity analysis

Characteristics of an eventual AIDS vaccine and of vaccination programs remain unknown, and assumptions were developed in order to provide estimates around which to base analyses (see Table 1). Assumptions are not based on any particular candidate in the current global pipeline. Our assumptions seek to carefully balance the potential promise of a vaccine against the remaining scientific challenges in AIDS vaccine research and development, the potential evolution of the broader HIV/AIDS response, the structural realities in relevant health care systems and the potential issues in sustainably reaching targeted populations, which may be further influenced by vaccine efficacy, required number of doses, costs, and duration of protection.

**Table 1:** Assumptions.

Parameter*	Base assumption	Variables for Sensitivity
**Introduction year**	2027	2025, 2030
**Efficacy**	70%	30, 40, 50, 60, 80, 90
**Doses (primary vaccination)**	3	2, 4
**Booster vaccination**	5 years (returning individual to full modeled efficacy for life)	3, 10, none necessary (lifetime immunity)
**Target coverage–generalized epidemics**	• Routine: 10 year olds: 70%	+/- 10, 20% for each tier
	• Catch up: 11–14 year olds: 60%	
	• Catch up: 15–17 year olds: 55%	
	• Catch up: 18–49 year olds: 50%	
**Target coverage–high risk populations in concentrated epidemics**	50%	30, 70
**Cost per regimen**	• LMICs: US $20 (US $5/dose + US $5 implementation)	See Table 2
	• MICs: US $55 (US $15/dose + US $10 implementation)	
**Rate at which target coverage is met (both epidemics)**	6 years	5, 10

*These parameters are illustrative and not tied to any specific vaccine candidate under current development.

Assumptions were developed by drawing on internal IAVI perspectives (including robust industry experience in launching new vaccines) and advice from experts in modeling, vaccine marketing and delivery, public health, and HIV/AIDS programs, including Gavi, the Vaccine Alliance, Global Fund to Fights AIDS, TB, and Malaria, Wits Reproductive Health and HIV Institute in South Africa, Bill and Melinda Gates Foundation, HIV prevention and vaccine researchers in sub-Saharan Africa, including KAVI Institute of Clinical Research, Uganda Virus Research Institute, and Aurum Institute in South Africa, and vaccine companies such as Merck, Sanofi Pasteur, Sanofi Pasteur MSD, and GlaxoSmithKline. A review of existing literature on uptake, compliance and cost of other vaccination programs and HIV/AIDS interventions as proxies further informed assumptions.

This study focused on a single base-case vaccine scenario instead of high/low scenarios used in the UNAIDS IFE in order to allow for independent variation of individual characteristics for focused interpretation of observed results. These sensitivity analyses covered a wide range of assumptions for individual product and program characteristics while maintaining other base case assumptions constant. However, we did not conduct comprehensive sensitivity or uncertainty analyses varying several parameters at the same time to explore the potential impact of interdependencies because of the large number of parameters and country simulations involved. High or low limits are not meant to represent the authors’ prediction of thresholds that regulatory agencies may require for licensure. Varying parameters in a wide range enables comparisons of which characteristics most strongly correlated to impact and cost-effectiveness (Table 1) and also provides a context similar to the use of high and low scenarios in the UNAIDS IFE.

The ALVAC and AIDSVAX HIV vaccine candidates reduced new infections by 60% after one year of follow-up and by 31% after three years of follow-up in the RV144 trial [19]. The illustrative base efficacy assumption of 70% efficacy in this modeling study assumes scientific innovation and progress will help enhance and sustain efficacy of future AIDS vaccines, and is consistent with optimistic goals for AIDS vaccine target product profiles [20]. Although the number of late-stage clinical trials remains low, the AIDS vaccine research field has incorporated the lessons of past challenges and has maintained a diversified product pipeline that holds the promise to produce an efficacious vaccine acceptable to regulators and policymakers as well as to the populations most in need. Nevertheless, we varied vaccine efficacy between 30% and 90% in sensitivity analysis. (In the model, 70% efficacy means that 100% of those vaccinated receive a 70% reduction in susceptibility to HIV infection.) We modeled reduced susceptibility to infection but not reduced transmission. The model further assumes a vaccine’s efficacy is sustained for the entirety of the durations modeled (five years for base case; three years, ten years and life-long in sensitivity analysis), but note that that modeling analyses of AIDS vaccines of waning immunity have previously been performed [21]. We further assume that vaccine efficacy is similar across different subtypes of HIV prevalent in different regions and epidemic types but acknowledge this as a limitation.

The base assumptions of three doses with a booster every five years seek a balance between complex vaccination regimens tested in both past and forthcoming AIDS vaccine clinical trials with strong recommendations from vaccination and public health experts that an AIDS vaccine regimen should be short, simple, and of minimal booster doses to help ensure strong acceptability and high levels of uptake and compliance with all required doses, including boosters, based on experience with other vaccination programs (HPV vaccination) and with other HIV/AIDS prevention or treatment options in reaching the target populations. We assumed constant coverage rates across all three doses of primary vaccination and booster doses. However, experience from other vaccinations and broad expert consensus suggest that compliance to AIDS vaccination will decrease with increasing numbers of primary doses required and also with the frequency of necessary booster vaccination, in particular in populations that are difficult to access due to structural, cultural, or social barriers. We explored the impact of these risks through lowering the coverage rate in our sensitivity analyses (see below) but we acknowledge that reality is more complex and that the influence of coverage rate and especially compliance to both primary and booster vaccination may be stronger than captured by our approximations.

Vaccine introduction in the base case of 2027, and in sensitivity analysis of 2025 and 2030 respectively, is consistent with the assumption of vaccine introduction in 2025 (high scenario) or 2030 (low scenario) in the UNAIDS IFE, and reflects optimism that candidates currently entering clinical development have the potential to move forward to licensure without significant delay. Further, recent mobilization for clinical testing of Ebola vaccine candidates supports optimism that enhanced international commitment could accelerate AIDS vaccine development significantly for promising candidates. While no AIDS vaccine candidates are currently being tested in preadolescents or adolescents, our assumptions incorporate optimism that an approach accepted for the more recently introduced HPV vaccine would be acceptable for AIDS vaccination. Upon demonstration of clinical efficacy in adults, licensure could occur rapidly based on relatively short bridging studies demonstrating immunogenicity in preadolescents or adolescents without clinical efficacy studies which would take much longer due to lower incidence, as well as greater size and complexity.

Coverage rates were assumed to differ depending on epidemic type. Base case coverage assumptions in generalized epidemics reflect optimism that high levels reached for other vaccination campaigns can also be achieved for AIDS vaccination. Target populations in generalized epidemics were seen to be analogous to those defined or projected for HPV vaccination, assuming strong delivery mechanisms and commitment from health authorities, ideally through school-based programs. In concentrated epidemics with high incidence in key specific populations, coverage of HIV/AIDS prevention, treatment, and care programs were seen as the primary proxy, acknowledging challenges in reaching such populations due to structural, social, and cultural barriers. Coverage sensitivity was calculated by taking base case coverage rates for generalized and concentrated epidemics and raising or lowering coverage by 10 or 20%.

The base assumption of six years for the rate at which target coverage is reached might also be considered optimistic, given historical lag in scaling up new vaccines [22]. However, a number of factors are likely to facilitate rapid AIDS vaccination scale-up, including high awareness of the severity of HIV/AIDS, existing infrastructure and sustained political support for HIV/AIDS treatment and prevention, as well as additional infrastructure from HPV vaccine programs targeting similar cohorts,. Further, the recent scale-up to almost total coverage levels in just a few years of meningococcal A vaccine in endemic African countries demonstrates how collaboration and strong planning can greatly accelerate vaccine coverage [23].

Further information on assumptions can be found in the Discussion and the S1 Appendix.

### Cost Scenarios

While the cost-of-goods and purchase price of a future AIDS vaccine remain unknown, a range of assumptions were formulated incorporating vaccine and implementation costs, which varied between LICs and MICs, in order to perform cost-effectiveness analyses (Table 2).

**Table 2:** Additional cost scenarios at launch.

	Doses	Low income countries (LMICs)			Middle income countries (MICs)		
		Per dose*	Implementation*	Total for Regimen*	Per dose*	Implementation*	Total*
**Launch scenarios**							
**Base**	3	5	5	20	15	10	55
**Higher price**	3	10	5	35	30	10	100
**Very high price**	3	20	5	65	50	10	160
**Middle income price = lower income price**	3	5	5	20	5	5	20
**2-dose regimen**	2	5	4	14	15	8	38
**4-dose regimen**	4	5	6	26	15	12	72
**10-years-out scenarios**							
**Base**	3	3	3	12	10	7	37
**Higher price**	3	5	3	18	15	7	52
**Very high price**	3	10	3	33	30	7	97
**Middle income price = lower income price**	3	3	3	12	3	3	12
**2-dose regimen**	2	3	2	8	10	5	25
**4-dose regimen**	4	3	4	16	10	9	49

*Costs presented in US$.

Cost scenarios are not representative of current candidates in development, but were formulated to provide a range for comparison and were influenced by available data for implementing existing vaccines, such as HPV, in LMICs [24]. This study assumes that tiered pricing for LICs and MICs will be utilized by industry, with LIC costs based on Gavi/UNICEF pricing for existing new vaccines. Additional information can be found in the S1 Appendix.

### Cost-effectiveness analyses

Quality-Adjusted Life Years (QALYs) measure the number of healthy years of life added by an intervention, e.g. a vaccine. Cost per QALY gained is a well-accepted measure to determine cost-effectiveness of that intervention in reducing disease burden. The WHO’s Commission on Macroeconomics and Health has classified interventions that gain a QALY at a cost that is less than a country’s Gross National Income (GNI) per capita as highly cost-effective, and interventions that cost between one and three times the GNI per capita as cost effective [25]. While the UNAIDS IFE aggregates individual epidemic models for LMICs, our model in some cases separates data for LICs and MICs to simulate differentiated considerations based on significantly different income level, corresponding to Gavi eligibility criteria. In this model, US$ 1,557 represents the average GNI per capita across LICs, based on purchasing power parity per capita (current international dollars) for LICs from the World Bank for 2013 (data were accessed on 10/12/2014).

## Results

Results of the model provide detailed estimates of the potential health impact, costs, and cost-effectiveness of AIDS vaccination in reducing new annual HIV infections and AIDS-related deaths in LMICs between an illustrative vaccine launch in 2027 and the year 2070. The following descriptions focus on the impact on new infections and cost-effectiveness; data on AIDS-related deaths are available but not shown. All figures are rounded since intrinsic uncertainties of the assumptions limit precision.

### Impact of AIDS vaccination on the number of new HIV infections in LMICs

**Fig 1:**
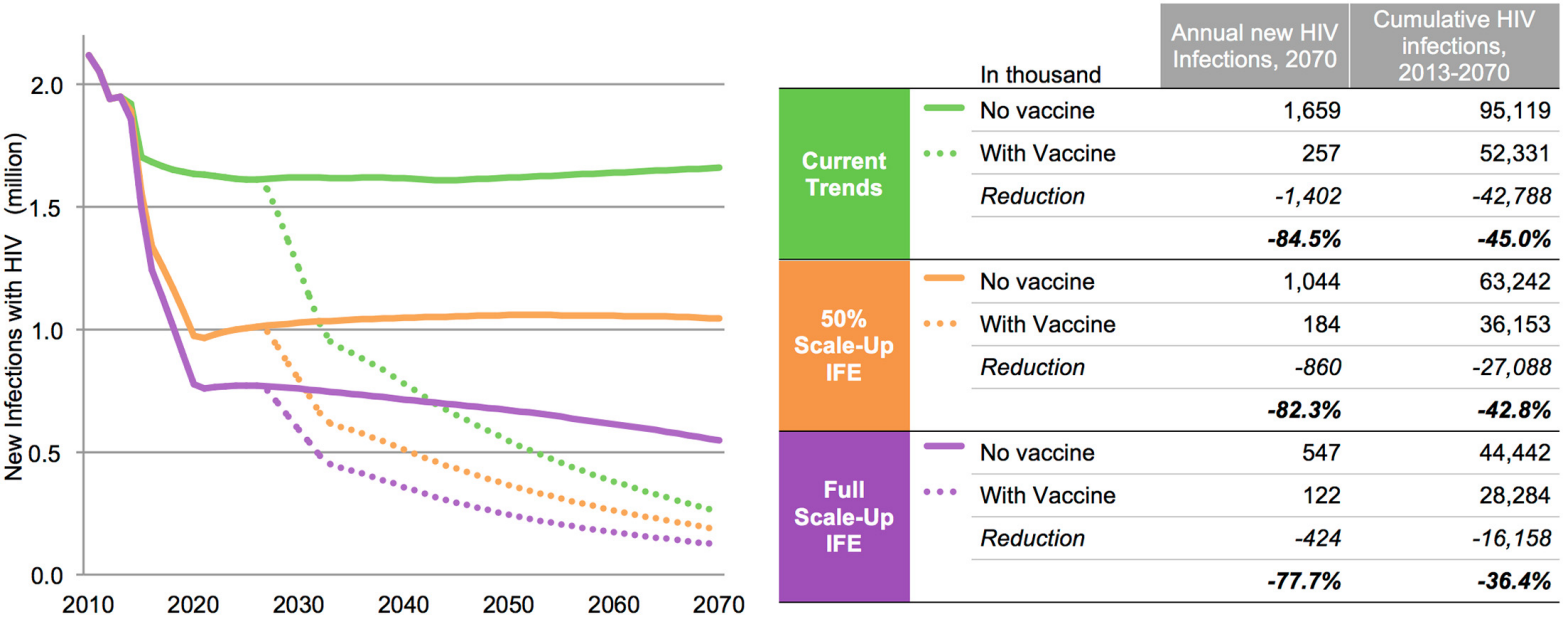
Reduction of new annual HIV infections with and without an AIDS vaccine under different IFE scale-up scenarios between 2010 and 2070. Vaccine and implementation characteristics are outlined in Table 1.

In the *Current Trends* scenario, incremental scale-up of existing HIV/AIDS interventions results in an eventual flat trajectory of new infections of around 1.6 million annually in 2070 (Fig 1). *Full Scale-up of IFE* and *50% Scale-up of IFE* would reduce the number of new annual HIV infections in 2070 to approximately 550,000 and 1 million, respectively. Including an AIDS vaccine (base-case characteristics, see Table 1) would reduce the annual number of new HIV infections in 2070 by 85% to around 260,000, by 78% to around 122,000, by 82% to around 184,000 when implemented with *Current Trends*, *Full Scale-up of IFE*, and *50% Scale-up of IFE*, respectively. Overall, an AIDS vaccine would reduce the number of cumulative HIV infections from 2027 to 2070 by more than 42 million, 16 million, and 27 million if applied to *Current Trends*, *Full Scale-up of IFE*, and *50% Scale-up of IFE*, respectively (Fig 1).

Unless noted, the following results on new infections refer to *Full Scale-up of IFE* as the fundamental background scenario. Vaccine impact is higher in *50% Scale-up of IFE* and *Current Trends* scenarios due to the higher incidence when a vaccine is introduced, but the relative proportion of new infections averted is relatively consistent with the *Full Scale-up of IFE* scenario (data not shown).

**Fig 2:**
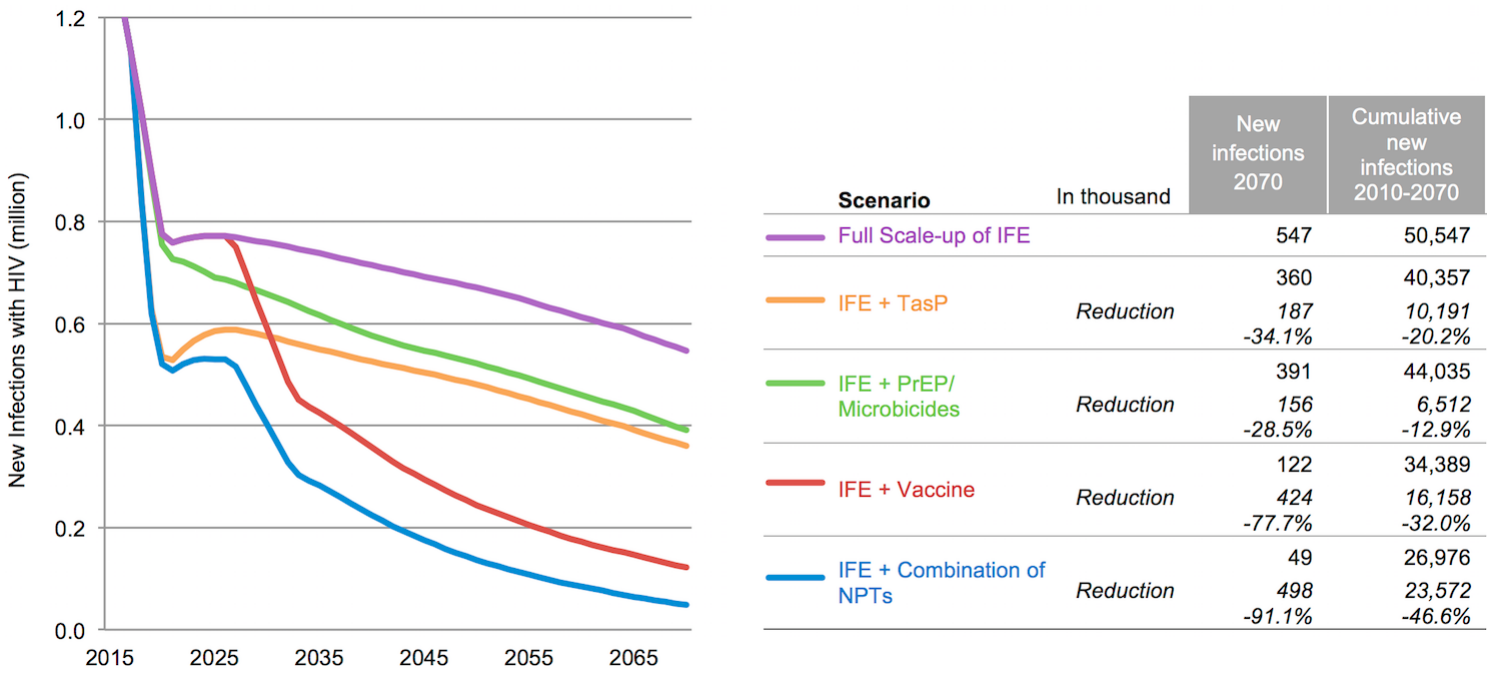
Reduction of new annual HIV infections with PrEP, TasP and vaccination added individually and in combination to the *Full Scale-up of IFE* between 2015 and 2070. Vaccine and implementation characteristics are outlined in Table 1. Assumptions on PrEP and TasP can be found in Stover et al. 2014 [14].

Adding PrEP, TasP, and an AIDS vaccine individually or in combination to the *Full Scale-up of IFE* reduces the number of annual new HIV infections in 2070 by 29%, 34%, 78%, and 91% respectively, with vaccination under base assumptions providing the strongest single benefit despite being introduced at a later stage. The combination of PrEP, TasP, and vaccination could reduce the number of annual infections with HIV to around 49,000 in 2070 (Fig 2). Assumptions for PrEP and TasP scale-up are detailed in Stover et al. 2014.

### Sensitivity analyses

#### Vaccine efficacy

**Fig 3:**
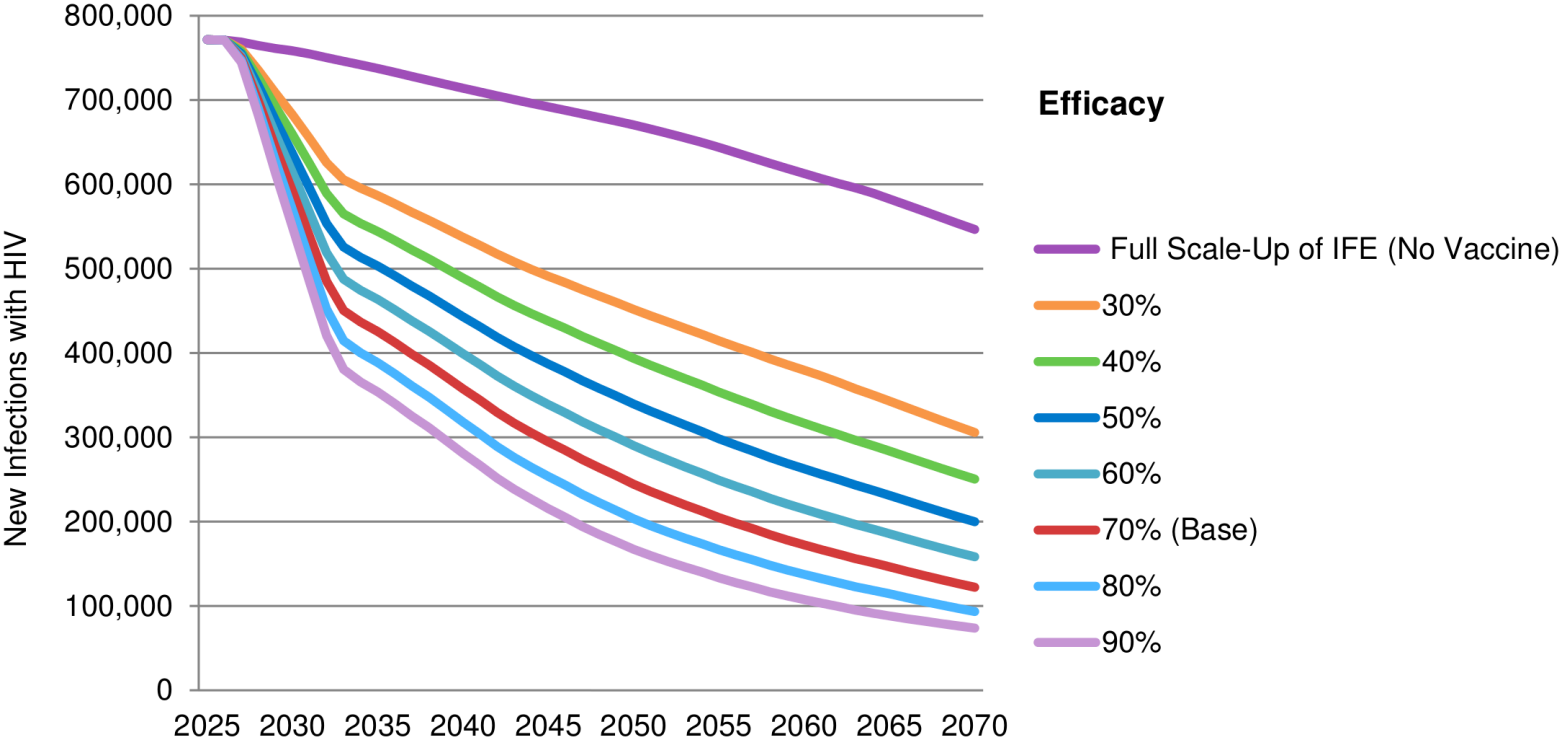
Reduction of new annual HIV infections under *Full Scale-up of IFE* according to vaccine efficacy between 2025 and 2070 (vaccine introduced in 2027). Other vaccine and implementation characteristics are outlined in Table 1.

There is strong correlation between vaccine efficacy and reduction of new annual HIV infections (Fig 3, Table 3). While a 70% efficacious vaccine (base-case) would reduce annual new infections by 78% to around 122,000 in 2070 when added to the *Full Scale-up of IFE*, a 30% efficacious vaccine would reduce new infections by 44% to 306,000 in 2070, and at 90% efficacy the reduction would be 87% to 74,000 new infections in 2070 (Fig 3).

**Table 3:** Potential new HIV infections averted in LMICs by vaccine efficacy. Other vaccine and implementation characteristics are outlined in Table 1.

Vaccine efficacy	Cumulative number of new HIV infections averted (2027–2070)
**30%**	8.4 million
**40%**	10.6 million
**50%**	12.6 million
**60%**	14.5 million
**70% (baseline)**	16.1 million
**80%**	17.7 million
**90%**	19.0 million

#### Coverage rate

**Fig 4:**
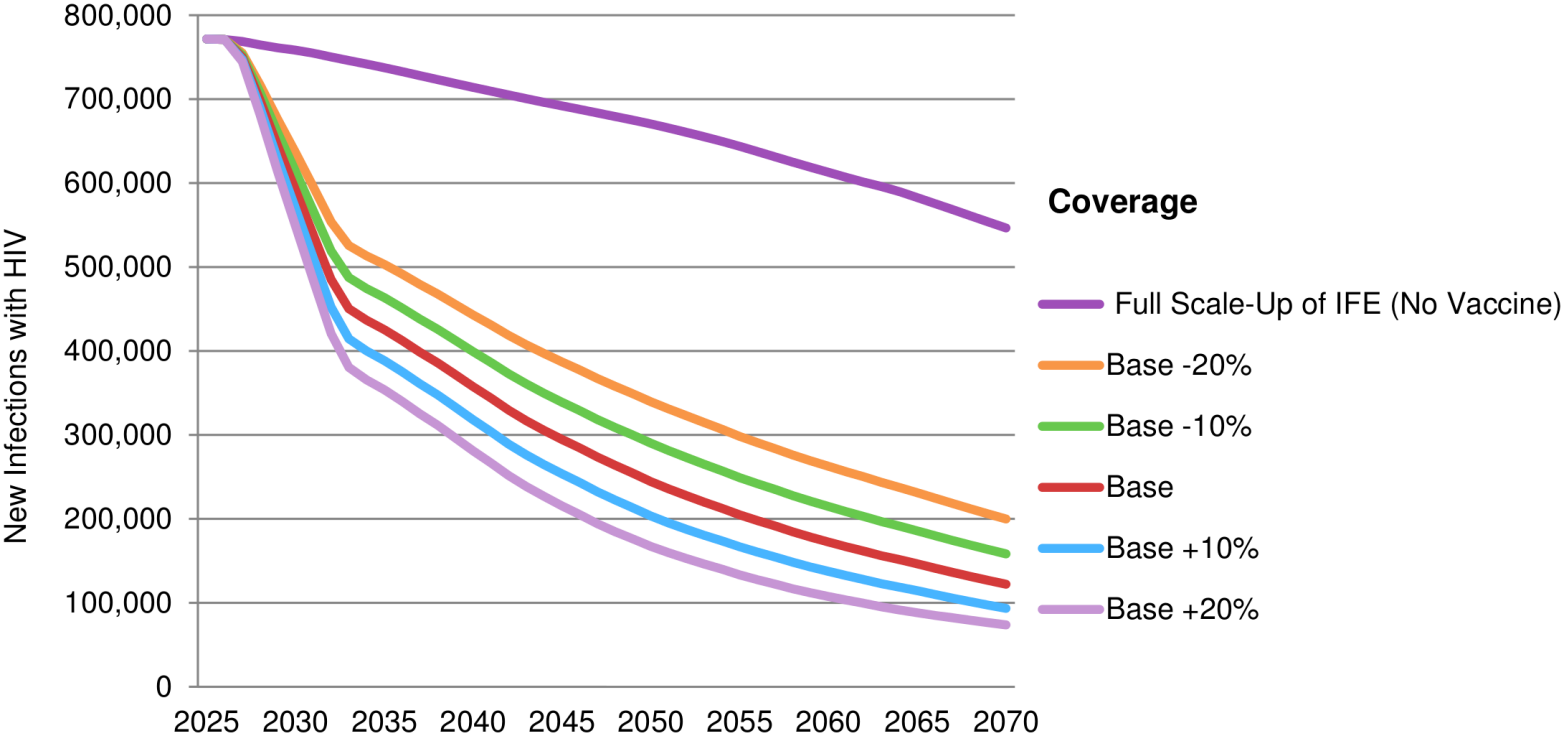
Reduction of new annual HIV infections under *Full Scale-up of IFE* according to vaccine uptake between 2025 and 2070 (vaccine introduced in 2027). Other vaccine and implementation characteristics are outlined in Table 1.

Higher vaccine uptake correlates to more new HIV infections averted. While under base-case assumptions new infections would be reduced by 78% to 122,000 in 2070, when added in 2027 to the *Full Scale-up of IFE* there would be 188,000 (66% fewer) and 225,000 (59% fewer) new annual infections in 2070 if the coverage rate was 10% or 20% lower than the base case. If uptake was 10% or 20% above base-case scenario there would be 74,000 (87% fewer) and 62,000 (89% fewer) new annual infections, respectively (Fig 4). The cumulative numbers of new annual HIV infections averted in LMICs through an AIDS vaccine by 2070 depending on coverage rate are presented in Table 4.

**Table 4:** Potential new HIV infections averted in LMICs by vaccine uptake.

Target coverage	Cumulative number of new HIV infections averted (2027–2070)
**-20%**	11.2 million
**-10%**	13.1 million
**Base coverage (see** Table 1**)**	16.2 million
**+10%**	18.7 million
**+20%**	20.0 million

### Cost-effectiveness

#### Cost-effectiveness related to cost per regimen

**Fig 5:**
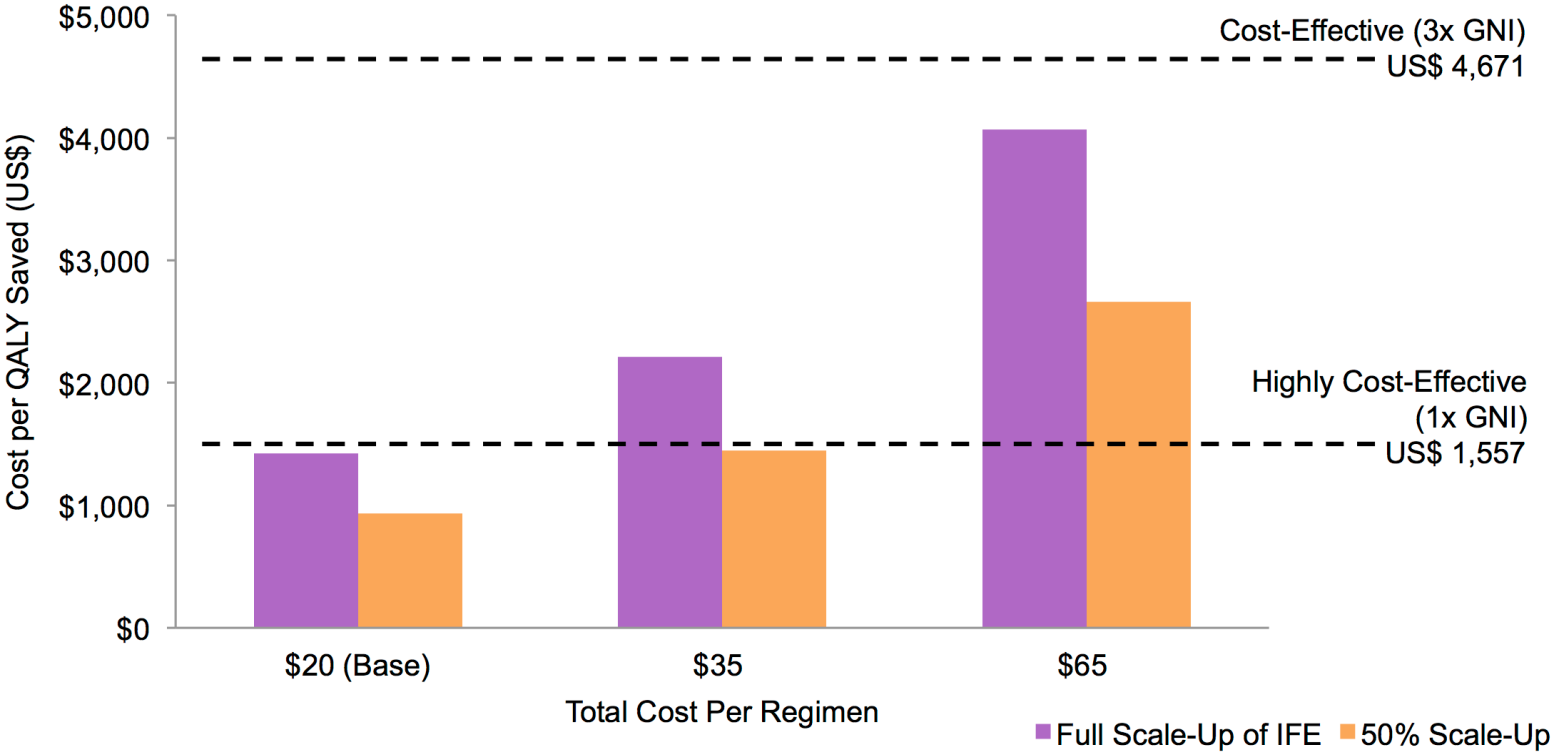
Cost per QALY gained with an AIDS vaccine in LICs added to *Full Scale-up of IFE* and *50% Scale-up of IFE* (discounted at 3% per year). Vaccine and implementation characteristics (base case) are outlined in Table 1.

In the base case scenarios looking at variable costs for a three-dose regimen, all cost scenarios analyzed are cost-effective (≤3x GNI per capita or US $4,671) in LICs under both *Full Scale-up of IFE* and *50% Scale-up of IFE*. Fig 5 shows that an AIDS vaccine in LICs would need to cost below US $20–25 per regimen under *Full Scale-up of IFE* and US $35–40 per regimen under *50% Scale-up of IFE* to be considered highly cost-effective (≤1x GNI per capita or US $1,557).

All the following results on cost-effectiveness refer to *Full Scale-up of IFE* as the fundamental scenario. Results are more favorable under *50% Scale-up of IFE* and *Current Trends* scenarios (see S1 Appendix).

#### Cost-effectiveness related to vaccine efficacy

**Fig 6:**
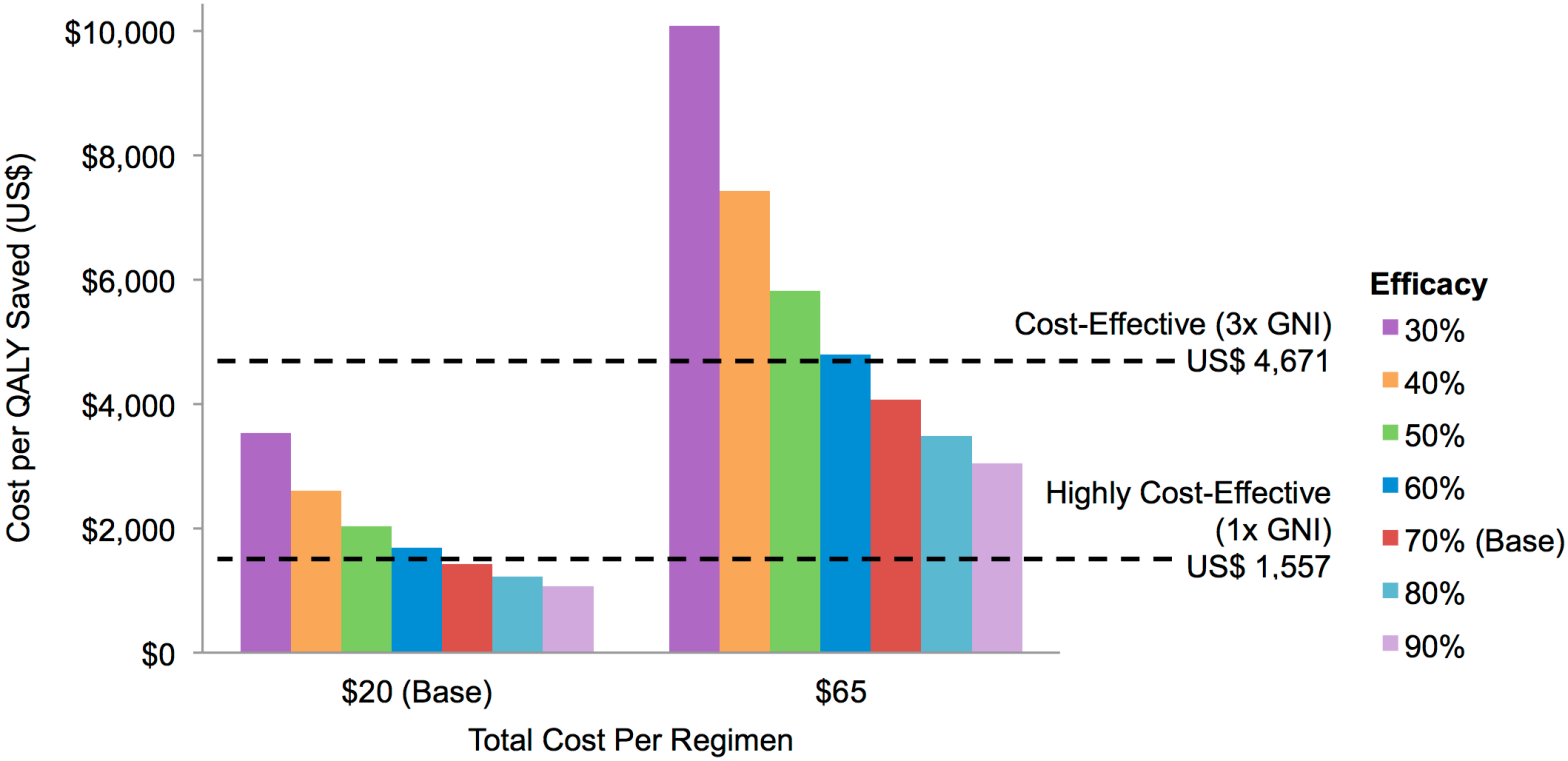
Cost per QALY gained (2027–2070) according to vaccine efficacy under two cost scenarios in LICs (discounted at 3% per year) when a vaccine is added to *Full Scale-up of IFE*. Other vaccine and implementation characteristics (base case) are outlined in Table 1. For cost assumptions see Table 2.

Focusing on LICs, Fig 6 illustrates that, at base case cost (US $20 per regimen), an AIDS vaccine would be cost-effective (≤3x GNI per capita or US $4,671) under all efficacy sensitivity scenarios (30–90%), and would be highly cost-effective (≤1 GNI per capita or US $1,557) when added to the *Full Scale-up of IFE* as long as the vaccine showed at least 60% efficacy. Under the very high price scenario (US $65 per regimen), an AIDS vaccine would need to be at least 60% efficacious to be cost-effective in LICs and would not be highly cost-effective at any of the assumed efficacy levels. Vaccines of all efficacy and cost levels modeled were cost-effective in MICs, with most also being highly cost-effective (see S1 Appendix).

#### Cost-effectiveness related to vaccine coverage

**Fig 7:**
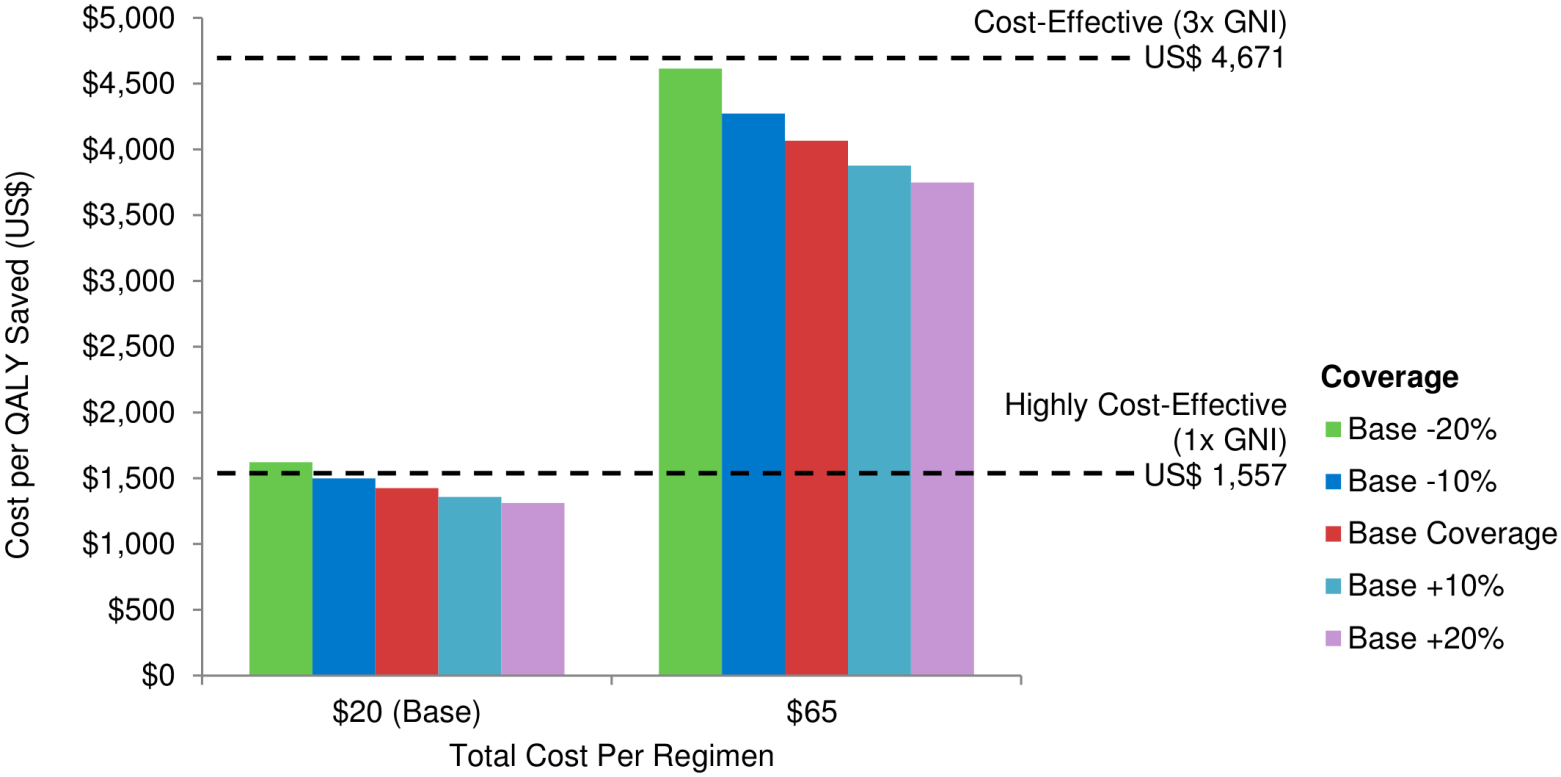
Cost per QALY gained (2027–2070) according to vaccine coverage under two cost scenarios in LICs (discounted at 3% per year) when a vaccine is added to *Full Scale-up of IFE*. Other vaccine and implementation characteristics (base case) are outlined in Table 1. For cost assumptions see Table 2.

Fig 7 illustrates that, at base coverage assumptions (see Table 1), an AIDS vaccine would be cost-effective (≤3x GNI per capita or US $4,671) in LICs under all coverage sensitivity scenarios and would be highly cost-effective or almost highly cost-effective (≤1 GNI per capita or US $1,557) when added to the *Full Scale-up of IFE*.

#### Cost-effectiveness related to duration of protection

A vaccine with base-case characteristics is cost-effective in LICs under almost all durations of protection analyzed, with only a vaccine under the *Very High Price* assumption and only 3 years duration not achieving cost-effectiveness. A vaccine of lifetime duration is highly cost-effective in all scenarios within the range of costs analyzed. Given the significantly higher GNI per capita in MICs (US $4,671), vaccination would be highly cost-effective at all assumed durations of protection despite being modeled at significantly higher costs than those in LICs (see S1 Appendix).

### Total costs of AIDS vaccination

**Fig 8:**
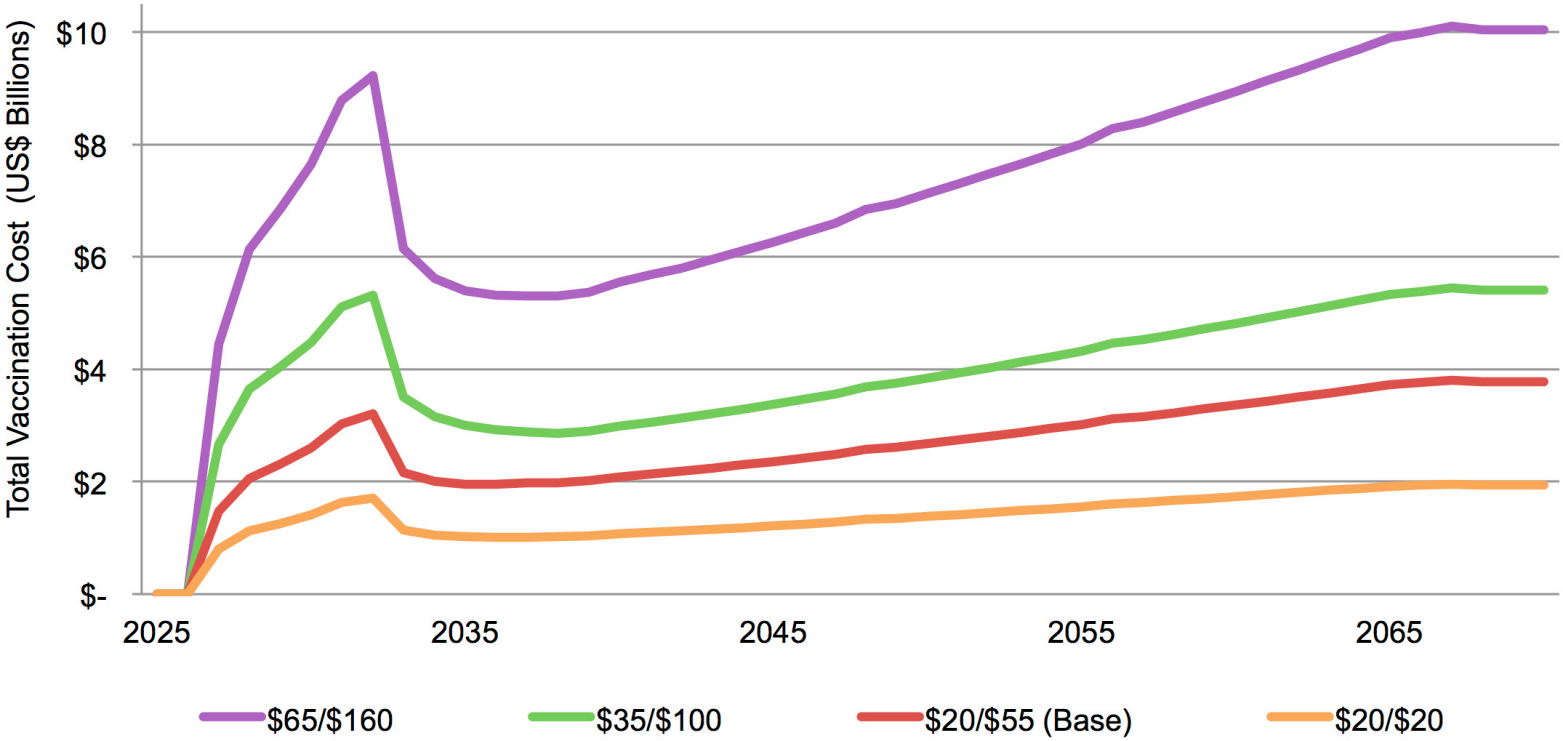
Estimated total costs for an AIDS vaccination program with base-case characteristics in LMICs under different cost-per-regimen scenarios (see Table 2). Base-case vaccine and implementation characteristics are outlined in Table 1.

The estimated total annual costs for implementing an AIDS vaccination program (with base-case vaccine characteristics, see Table 1) in LMICs under different cost-per-regimen scenarios (see Table 2) within the *Full Scale-up of IFE* are explored in Fig 8. During the catch-up phase the total annual costs spike to US $3.2 billion before stabilizing at a range of US $1 billion (cost per regimen of US $20/US $20 in LICs/MICs) to US $5 billion (cost per regimen of US $65/US $160 in LICs/MICs) per year. Over time, the annual vaccination cost increases, which reflects an increase in the total number of AIDS vaccinations needed to maintain target coverage as populations increase.

**Fig 9:**
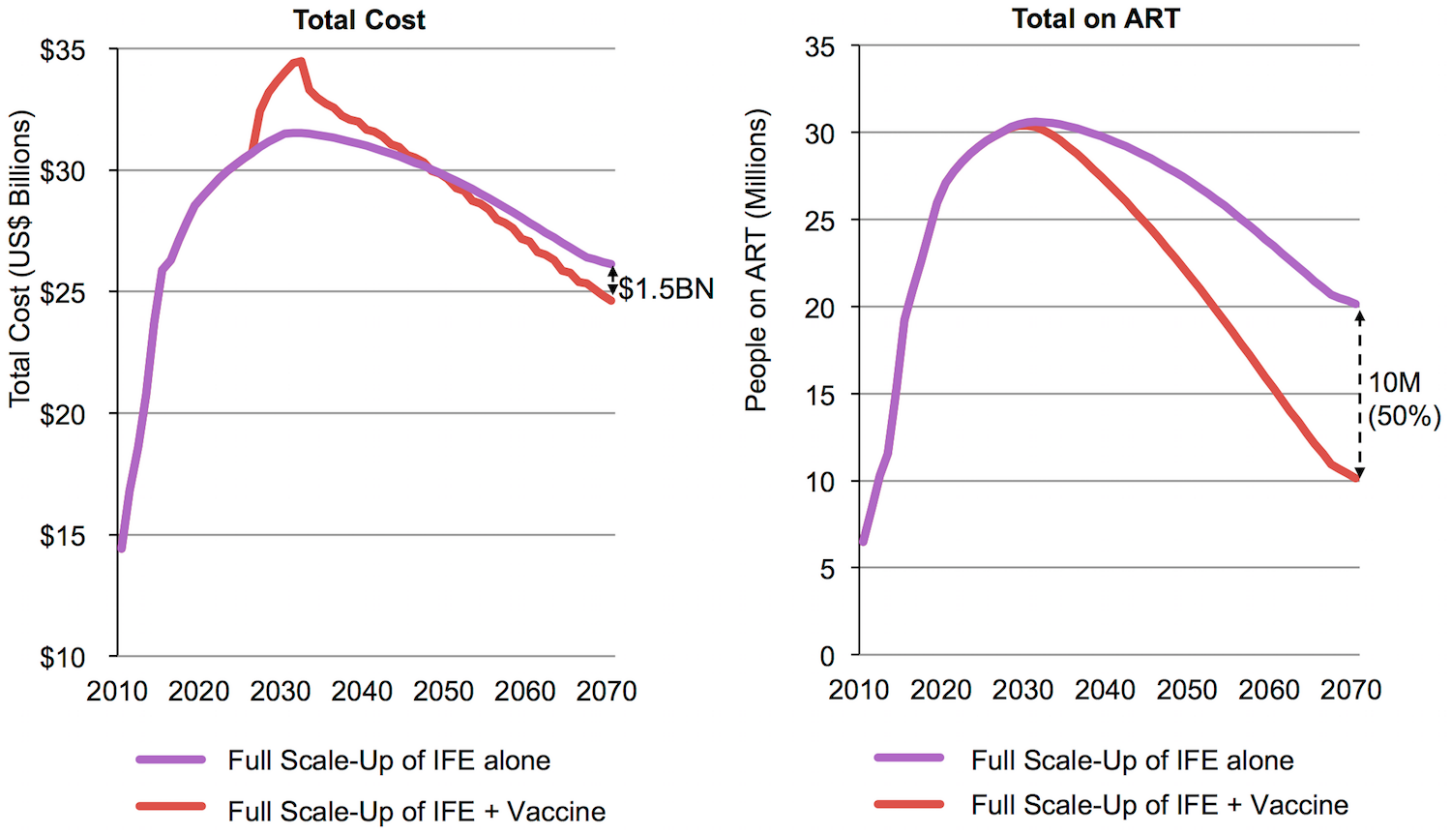
Costs for both *Full Scale-up of IFE* as a whole and the ART component with this scale-up and number of people on antiretroviral treatment both with and without AIDS vaccination (base case-characteristics). Base-case vaccine and implementation characteristics are outlined in Table 1. For base-case cost assumptions see Table 2.

Fig 9 projects costs for both *Full-scale-up of IFE* alone and *Full Scale-up of IFE* plus vaccine (left), as well as the total number of individuals accessing ART within *Full Scale-up of IFE* in scenarios both with and without the addition of a vaccine (right, base-case characteristics for both, see Table 1). This assumes all existing interventions continue at *Full Scale-up of IFE* levels after introduction of an AIDS vaccine. Adding an AIDS vaccine, with temporary catch-up vaccination, results in a spike in total costs of approximately US $3–4 billion, before total costs start decreasing as of 2048 thanks to a long-term reduction (50%) in the number of individuals requiring ART from 20 million to 10 million as new infections are averted by vaccination. By 2070, US $1.5 billion would be saved per year. Cumulatively, adding an AIDS vaccine to the IFE would require a net investment of US $9 billion between 2027 and 2070.

## Discussion

While the accuracy of the presented results is limited by the inherent uncertainties of both the model itself and the underlying assumptions, these results highlight the significant value of an AIDS vaccine under a variety of model scenarios and vaccine characteristics. Sensitivity analyses varying individual characteristics in wide ranges with other variables remaining constant explored the relative contribution of those characteristics. While these analyses do not replace comprehensive uncertainty analyses to explore the potential for one or more of those characteristics to influence another (e.g. lower efficacy and duration of protection or increasing number of doses potentially lowering vaccine uptake and compliance with multiple doses), they illustrate the potential impact of less or more optimistic assumptions on the potential health impact and cost-effectiveness of future AIDS vaccination.

The *High* and *Low* vaccine scenarios utilized in the UNAIDS IFE study provide some additional context in regard to the potential impact of varying combined assumptions [14]. Added to a baseline IFE scenario, the *High* vaccine scenario further reduces annual new infections by 75% from 1.1 million to 140,000 by 2050, and the *Low* vaccine scenario further reduces annual new infections by 37% to 340,00. Finally, by largely focusing on *Full Scale-up of IFE* scenario as the primary baseline for our results, we provide a conservative set of impact and cost-effectiveness data for a vaccine assuming the optimistic assumptions of the UNAIDS IFE for all other interventions are fully achieved.

This analysis does not purport to provide a perfect prediction of either a vaccine’s impact or of the effectiveness of future AIDS vaccination programs. However, we are confident that these results provide valuable context to guide necessary decisions pertaining to the research and development of future AIDS vaccines, its funding, and eventually its delivery.

The data suggest that under certain circumstances vaccination could prevent more new infections with HIV than other new prevention options. However, the modeling also confirms that no single option can solve the problem alone. A variety of prevention and treatment options can complement each other in ensuring that the specific needs of specific populations in different circumstances are met in order to maximize the reduction of new HIV infections. Further, lessons learned in the implementation of other prevention options can help maximize the success of future AIDS vaccination.

This modeling shows that vaccine impact will depend significantly on vaccine efficacy and vaccine coverage. First generation AIDS vaccines may not achieve the very high efficacy levels of most other currently licensed vaccines, and structural, cultural, and social barriers may make it challenging to meet assumed coverage rates for some target populations, but the model shows that vaccines of relatively lower efficacy and uptake still have the potential to substantially reduce new HIV infections.

Vaccine cost-effectiveness is strongly linked to cost per regimen, efficacy, coverage, and duration of protection. Cost-effectiveness is a major consideration by policymakers and implementers of vaccine programs, in particular when healthcare budgets are constrained. Domestic health budgets and donor funding for health in LMICs should consider cost-effectiveness in decisions on rationally allocating limited health resources toward interventions across a wide spectrum of health issues [26]. Further, Gavi eligibility is based on a GNI per capita below or equal to US $1,580 [27]. We therefore feel strongly that meeting the very similar highly cost effective threshold by WHO’s Commission on Macroeconomics and Health (US $1,557) will be important toward ensuring accessibility to future AIDS vaccines, especially in LICs. In this modeling a vaccine meets the “highly cost effective” threshold in LICs only at the lower costs modeled whereas a vaccine of base-case characteristics would be highly cost-effective under even very high cost assumptions in MICs. However, it should be noted that our model does not capture future increases of GNI per capita in LMICs and the resulting shifts in thresholds for Gavi eligibility. Nevertheless, reducing the number of doses and increasing the duration of protection both increase cost-effectiveness.

Estimated annual spending on the HIV/AIDS response in LMICs totaled US $20.2 billion in 2014 [28]. Resource needs for the UNAIDS IF were estimated at US $22–24 billion annually and US $31.5 billion per year for the UNAIDS IFE [13, 14]. Estimates of necessary resources to meet UNAIDS’ more recent 90/90/90 treatment-focused targets and the prevention and non-discrimination targets could to reach over US $31 billion annually [7]. This model assumes that the introduction of an AIDS vaccine would not replace other prevention methods but would instead provide an addition to the comprehensive response. However, fewer new infections would result in a reduced number of people living with HIV and in turn reductions in overall treatment costs, with annual savings eventually outpacing annual costs of vaccination programs. Nevertheless, when considering cumulative cost, implementing AIDS vaccination would require an investment. This additional investment should be seen in light of the sizeable potential health impact in terms of reduced new infections with HIV and in turn less AIDS-related deaths and human suffering, and in turn a reduction of the obstacles to societal and economic development HIV/AIDS represents to the most affected countries. Finally, our modeling of total vaccination cost and total cost of the comprehensive HIV/AIDS response in the UNAIDS IFE through 2070 did not consider that more targeted and less costly vaccination and other prevention programs may be warranted with decreasing HIV incidence and correlating reductions in total treatment cost. While predicting such effects with accuracy is difficult, comprehensive combination prevention programs have the potential to reduce generalized epidemics to smaller concentrated ones, although the risk remains within these concentrations for the re-emergence of high incidence. Prior exercises have shown the potential to achieve efficiencies and reduce vaccination costs with relatively small impact on reductions in new infections [29], signaling that cost savings from a vaccine could be higher than the projected US$1.5 billion per year in 2070.

## Conclusions

The UNAIDS IFE provides a pathway to continued progress in the fight against HIV/AIDS, but the achievement of these targets will continue to be challenged by resource and capacity limitations, as well as structural, social, and cultural barriers in reaching most-affected populations. These projections show that a combined approach scaling up existing HIV/AIDS prevention, treatment, and care programs in LMICs while incorporating new prevention options, including a vaccine, provides the greatest benefit in terms of reduced new infections, and that a vaccine can play a critical role in the comprehensive response toward a sustainable end to AIDS.

A modestly effective vaccine would reduce new infections significantly and be cost-effective, even if other interventions reach optimistic coverage targets. A highly effective vaccine would provide even greater impact and cost-effectiveness, and would support broader access. Cost per regimen, efficacy, coverage, and duration of protection are critical factors to achieve acceptable cost-effectiveness, particularly in low-income countries. Fewer doses per regimen and longer duration of protection significantly reduce total vaccination cost. A vaccine could significantly reduce treatment cost and even be cost-saving over time. An optimal mix of vaccine characteristics will be critical to ensuring that a vaccine is acceptable and accessible globally, even in different epidemic types.

## Supporting Information

S1 Appendix Exploring the potential health impact and cost-effectiveness of adding an AIDS vaccine to the enhanced UNAIDS Investment Framework: Web Appendix. PDF document providing additional information on assumption rationale and expanded datasets mentioned in the main text.
(DOCX)

## References

[1] UNAIDS. How AIDS Changed Everything: MDG 6: 15 Years, 15 Lessons of Hope from the AIDS Response. http://www.unaids.org: 2015 7 2015.

[2] UNAIDS. AIDSinfo: Epidemiological status: UNAIDS; 2014 [5 October 2015]. Available from: http://www.unaids.org/en/dataanalysis/datatools/aidsinfo.

[3] Pruss-Ustun A, Wolf J, Driscoll T, Degenhardt L, Neira M, Calleja JM. HIV due to female sex work: regional and global estimates. PLoS One. 2013;8(5):e63476 10.1371/journal.pone.0063476 23717432 PMC3662690

[4] Cohen MS, Chen YQ, McCauley M, Gamble T, Hosseinipour MC, Kumarasamy N, et al Prevention of HIV-1 infection with early antiretroviral therapy. N Engl J Med. 2011;365(6):493–505. 10.1056/NEJMoa1105243 21767103 PMC3200068

[5] Raymond A, Hill A, Pozniak A. Large disparities in HIV treatment cascades between eight European and high-income countries–analysis of break points. Journal of the International AIDS Society. 2014, 17(Suppl 3):19507 25394016 10.7448/IAS.17.4.19507 PMC4224795

[6] World Health Organization. Guideline on when to start antiretroviral therapy and on pre-exposure prophylaxis for HIV.www.who.int. 9 2015. 26598776

[7] UNAIDS. On the Fast-Track to end AIDS by 2030—Focus on location and population (World AIDS Day Report 2015). http://www.unaids.org November 30.

[8] Esparza J. A brief history of the global effort to develop a preventive HIV vaccine. Vaccine. 2013;31(35):3502–18. 10.1016/j.vaccine.2013.05.018 23707164

[9] Lema D, Garcia A, De Sanctis JB. HIV vaccines: a brief overview. Scand J Immunol. 2014;80(1):1–11. 10.1111/sji.12184 24813074

[10] Rerks-Ngarm S, Pitisuttithum P, Nitayaphan S, Kaewkungwal J, Chiu J, Paris R, et al Vaccination with ALVAC and AIDSVAX to prevent HIV-1 infection in Thailand. N Engl J Med. 2009;361(23):2209–20. 10.1056/NEJMoa0908492 19843557

[11] National Institute of Allergy and Infectious Diseases. NIH-sponsored HIV vaccine trial launches in South Africa: Early-stage trial aims to build on RV144 results: http://www.nlaid.nih.giv; 2015 [updated 18 February 210523 June 2015]. Available from: http://www.niaid.nih.gov/news/newsreleases/2015/Pages/HVTN100.aspx.

[12] IAVIReport. Clinical Trials Database: Database of vaccine candidates in clinical trials: IAVIReport; 2015 [23 June 2015]. Available from: http://www.iavireport.org/Trials-Database/Pages/default.aspx.

[13] Schwartländer B, Stover J, Hallett T, Atun R, Avila C, Gouws E, et al Towards an improved investment approach for an effective response to HIV/AIDS. Lancet. 2011;377(9782):2031–2041. 10.1016/S0140-6736(11)60702-2 21641026

[14] Stover J, Hallett TB, Wu Z, Warren M, Gopalappa C, Pretorius C, et al How can we get close to zero? The potential contribution of biomedical prevention and the investment framework towards an effective response to HIV. PLoS One 9(11), e111956, L2014. 10.1371/journal.pone.0111956 25372770 PMC4221192

[15] Available via Avenir Institute. http://www.avenirhealth.org/software-spectrummodels.php

[16] Stover J, Bollinger L, Hecht R, Williams C, Roca E. The impact of an AIDS vaccine in developing countries: a new model and initial results. Health Aff (Millwood). 2007;26(4):1147–58. 17630459 10.1377/hlthaff.26.4.1147

[17] Jit M, Brisson M, Laprise JF, Choi YH. Comparison of two dose and three dose human papillomavirus vaccine schedules: cost effectiveness analysis based on transmission model. BMJ. 2015;350:g7584 10.1136/bmj.g7584 25567037 PMC4285892

[18] International Association of Providers of AIDS Care. Global HIV Policy Watch: ART eligibility criteria for asymptomatic people living with HIV. [Updated: July 16, 2015] Available From: http://hivpolicywatch.org

[19] Kim JH, Rerks-Ngarm S, Excler JL, Michael NL. HIV vaccines: lessons learned and the way forward. Curr Opin HIV AIDS. 2010;5(5):428–34. 10.1097/COH.0b013e32833d17ac 20978385 PMC2990218

[20] AVAC. AIDS vaccine research: An overview: http://www.avac.org; 2015 [updated May 201523 June 2015]. Available from: http://www.avac.org/sites/default/files/event_files/HVAD2015_slides.pdf.

[21] Hankins CA, Glasser JW, Chen RT. Modeling the impact of RV144-like vaccines on HIV transmission. Vaccine. 2011;29(36):6069–71. 10.1016/j.vaccine.2011.07.001 21762753 PMC4580279

[22] Levine OS, Knoll MD, Jones A, Walker DG, Risko N, Gilani Z. Global status of Haemophilus influenzae type b and pneumococcal conjugate vaccines: evidence, policies, and introductions. Curr Opin Infect Dis. 2010;23(3):236–41. 10.1097/QCO.0b013e328338c135 20407316

[23] Kristiansen PA, Diomande F, Ba AK, Sanou I, Ouedraogo AS, Ouedraogo R, et al Impact of the serogroup A meningococcal conjugate vaccine, MenAfriVac, on carriage and herd immunity. Clin Infect Dis. 2013;56(3):354–63. 10.1093/cid/cis892 23087396

[24] Levin A, Wang SA, Levin C, Tsu V, Hutubessy R. Costs of introducing and delivering HPV vaccines in low and lower middle income countries: inputs for GAVI policy on introduction grant support to countries. PLoS One. 2014;9(6):e101114 10.1371/journal.pone.0101114 24968002 PMC4072768

[25] World Health Organization. Macroeconomics and Health: Investing in health for economic development. Report of the Commission on Macroeconomics and Health. 2001 20 December 2001. Report No.: 2001/13984.

[26] Newall AT, Jit M, Hutubessy R. Are current cost-effectiveness thresholds for low- and middle-income countries useful? Examples from the world of vaccines. Pharmacoeconomics. 2014;32(6):525–31. 10.1007/s40273-014-0162-x 24791735

[27] GAVI: The vaccine alliance. Countries eligible for support: http://www.gavi.org; 2015 [23 June 2015]. Available from: http://www.gavi.org/support/apply/countries-eligible-for-support/.

[28] The Henry J. Kaiser Family Foundation and UNAIDS. Financing the Response to HIV in Low- and Middle-Income Countries: International Assistance from Donor Governments in 2014. http://www.unaids.org: 2015 July 2015. Report No.: Contract No.: #7347–11.

[29] Harmon T, Guo W, Stover J, Wu Z, Kaufman J, Schneider K, et al The potential impact of preventive HIV vaccines in China: Results and benefits of a multi-province modeling collaboration. Vaccines. 2015;3(1):1–19. 10.3390/vaccines3010001 26344945 PMC4494240

