# Supplementary material for: Exploring the Potential Health Impact and Cost-Effectiveness of AIDS Vaccine within a Comprehensive HIV/AIDS Response in Low- and Middle-Income Countries
Source: PLoS One. 2016 Jan 5;11(1):e0146387. doi: 10.1371/journal.pone.0146387 (PMC4701445; doi:10.1371/journal.pone.0146387)
Supplement: S1 Appendix — PDF document providing additional information on assumption rationale and expanded datasets mentioned in the main text. (DOCX) [file pone.0146387.s001.docx]

**Exploring the Potential Health Impact and Cost-Effectiveness of AIDS Vaccine Within a Comprehensive HIV/AIDS Response in Low- and Middle-Income Countries**

***Modeling an AIDS Vaccine’s Impact within the Enhanced UNAIDS Investment Framework***

**Adjusted assumptions about the UNAIDS enhanced Investment Framework (IFE)**

Detailed UNAIDS IFE assumptions can be found in the background materials to Stover et al [^[[1]](#endnote-2)^]. For the purposes of this vaccine-specific publication, achievement of target levels for IFE interventions in low- and middle-income countries (LMICs) was delayed to 2020 to reflect challenges in achieving targets that have been observed since they were first proposed as part of the original 2011 Investment Framework. This has minimal impact on results for vaccine modeling and avoids an unrealistic drop in new HIV infections in the period between 2013 and 2015.

**Evolution of key assumptions of vaccine and vaccination characteristics**

High and Low scenario AIDS vaccine assumptions as utilized in Stover et al [i] were produced by representatives of the International AIDS Initiative (IAVI), the Bill and Melinda Gates Foundation (BMGF) and AVAC and endorsed by the UNAIDS Investment Framework New Prevention Technology (NPT) Working Group, and are summarized in Table S1.

**Table S1: Original Base AIDS Vaccine Scenarios – Stover et al [i].**

| **Scenario** | **Efficacy** | **Boosters** | **Intro Year** | **Year Target Coverage Achieved** | **Epidemic Type** | **Coverage** |
| --- | --- | --- | --- | --- | --- | --- |
| **High** | 80% | Every 10 years | 2025 | 2032 | Adult population in generalized epidemics | 70% |
|  |  |  |  |  | High-risk population in concentrated epidemics | 60% |
| **Low** | 60% | Every 3 years | 2030 | 2035 | Adult population in generalized epidemics | 40% |
|  |  |  |  |  | High-risk population in concentrated epidemics | 30% |

For this vaccine-specific paper we focus on a single base-case vaccine scenario instead of High/Low scenarios in the UNAIDS, in order to simplify messaging around vaccine impact and provide a simpler scenario on which to build independent sensitivity analyses for each parameter. While this represents a significant change from the IFE modeling and cannot replace comprehensive uncertainty analysis which aims to explore the potential impact of interdependent parameters, we favored independent sensitivity analyses to allow for independent observation of the potential impact of each parameter. We refer to High and Low scenarios in the UNAIDS IFE for some guidance on how the coupled variation of independent parameters could influence results. We also consider different vaccination costs for low-income countries (LICs) and middle-income countries (MICs) in order to better approximate real-world global vaccination programs.

**Table S2: Vaccine Characteristic Assumptions**

| Parameter* | Base Assumption | Variables for Sensitivity |
| --- | --- | --- |
| Introduction year | 2027 | 2025, 2030 |
| Efficacy | 70% | 30, 40, 50, 60, 80, 90 |
| Doses (primary vaccination) | 3 | 2, 4 |
| Booster vaccination | 5 years (returning individual to full modeled efficacy for life) | 3, 10, none necessary (lifetime immunity) |
| Target coverage – generalized epidemics | - Routine: 10 year olds: 70% - Catch up: 11–14 year olds: 60% - Catch up: 15–17 year olds: 55% - Catch up: 18–49 year olds: 50% | +/- 10, 20% for each tier |
| Target coverage – high risk populations in concentrated epidemics | 50% | 30, 70 |
| Cost per regimen | - LICs: US $20 (US $5/dose + US $5 implementation) - MICs: US $55 (US $15/dose + US $10 implementation) | See Table 2 |
| Rate at which target coverage is met (both epidemics) | 6 years | 5, 10 |

The following draws from the above sources to provide a summarized rationale for each proposed assumption.

**Vaccine Efficacy**

The RV144 phase III trial coordinated by the United States Military HIV Research Program and the government of Thailand that combined the ALVAC and AIDSVAX candidates provided the first signals of AIDS vaccine efficacy, reducing new infections by 31% [^[[2]](#endnote-3)^]. A vaccine of such efficacy would likely be unacceptable for a large-scale public health program [^[[3]](#endnote-4)^]. The High and Low scenarios of the UNAIDS IFE (Table S1) assumed a vaccine efficacy of 80% and 60%, respectively. One participant in expert consultations noted that an AIDS vaccine of less than 50% efficacy would be very challenging to implement as adoption and adherence would be low, although another respondent made the point that given the high burden of disease, even at low efficacy the public health impact of a vaccine could be high. Other experts pointed out that 60–70% efficacy would be an acceptable assumption for an AIDS vaccine in light of currently available data, but that 80% was too high. A base efficacy of 70% was chosen to provide balance between a highly optimistic, almost fully protective vaccine and the 50% efficacy that is seen by many as the lowest possible licensable product. Varying efficacy in the model from very low (30%) to very high (90%) in the sensitivity analysis provides an illustration of the relative importance of efficacy in the base case.

In the model, the vaccine’s efficacy is stable throughout; we assumed no waning effect. This was primarily done to maintain simplicity in the model, but is also in line with other modeling efforts, including one by a vaccine manufacturer on which IAVI is currently consulting. A series of articles in the journal *Vaccine* recently explored the potential impact of RV144-like vaccine regimens on new HIV infections and AIDS-related deaths under different models [^[[4]](#endnote-5)^].

**Introduction Year**

The timeline for the development, licensure, and delivery of an AIDS vaccine is difficult to predict. There are no current candidates in phase IIb or phase III trials [^[[5]](#endnote-6)^], although follow-up studies of the RV144 trial are expected to commence in South Africa in 2016 [^[[6]](#endnote-7)^].

A relatively optimistic introduction date of 2027 was modeled in the base case in order to balance optimism in current candidates moving forward and significant scientific challenges that remain to be overcome. Sensitivity analyses allow an exploration of how moving the timeline forward or backward several years relates to vaccine impact. The current Ebola crisis demonstrates how global sense of urgency and increased commitment could accelerate development. We assumed a similar acceleration should there be a major breakthrough in AIDS vaccine research and development.

**Number of doses – primary vaccination**

RV144 was a six-dose regimen, more doses than public health experts and experience with other vaccinations suggest to be realistic in regard to adherence, especially in low- and middle-income countries and for the vaccination of adolescents and adults outside of school-based programs. A number of consulted experts noted that anything more than three doses would be considered as unrealistic as a widely implemented public health tool, given that adherence will be too low, especially in adult populations. High adherence to the three-dose vaccine against human papillomavirus (HPV) is seen primarily in school-based programs, where vaccinations are tied to attendance [^[[7]](#endnote-8)^]. Data from countries with non-school-based vaccination show significantly decreasing adherence with an increasing number of doses.

A three-dose regimen was used in the base case, with sensitivity done for two and four doses in order to explore the cost implications of a regimen requiring fewer or more doses. Reduced efficacy for lower adherence was not modeled in order to maintain a level of simplicity in the model; however, the implications of low adherence can be explored as a variation on lower vaccine coverage, as discussed in sections below.

**Booster timing/effect**

The duration of protection from an AIDS vaccine is unknown. However, a longer duration would be ideal both to enhance adherence and to reduce cost. One expert noted that duration shorter than 5 years would be challenging to implement successfully as adherence to boosters would likely be low. Others were interested in how adherence to shorter-duration vaccines would influence vaccine impact, with one noting that duration is just as critical as efficacy in this regard. Duration was seen as an influencer on the age at which a vaccine would be administered, which is consistent with considerations by experts and governments prior to the introduction of HPV vaccination; a vaccine of short duration would make most sense in populations near to or after age of sexual debut in order to provide protection at time of first exposure to HIV.

A base case of a five-year duration was ultimately used, with sensitivity analyses of three-year, ten-year, and lifetime duration. Modeling various durations can inform public health decisions, exploring both impact and cost implications. Full compliance to all three doses was assumed in order to maintain relative simplicity in the model, but reduced adherence can be explored as a variation on reduced coverage, as discussed in sections below.

The model assumes that the booster maintains the vaccine’s original efficacy.

**Target coverage by age group: generalized epidemics**

In the original UNAIDS IFE High and Low scenarios, assumptions around coverage in generalized epidemics included all persons aged 15–49, and were quite high across those age groups in our view. Consultations with experts on assumptions for a more granular vaccine model encouraged considerations around the implementation of existing vaccines targeted to adults and adolescents, especially HPV vaccination. Such programs have met with challenges in achieving high coverage in adults to date, and while HPV vaccine demonstration programs have achieved rates of coverage of 80–90% [^[[8]](#endnote-9)^] (especially in school-aged programs), there was little data supporting rapid, wide-scale coverage across 35 complete age cohorts across adolescents and adults in LMICs, especially outside school-based vaccination or above school age. Relatively low coverage with influenza vaccines or DTP booster vaccination in adolescents even in developed countries was also cited [^[[9]](#endnote-10)^].

Further, in several cases, programs to implement relatively easier-to-deliver infant vaccines have not yet reached high levels in LMICs [^[[10]](#endnote-11)^]. It was noted that a vaccine of significantly less than 90% efficacy could prove even more difficult to implement and achieve high coverage. One expert especially noted that for a multi-dose vaccine, reaching full coverage with all necessary doses would be a massive challenge, saying that a three-dose vaccine would probably at best achieve 30% coverage of full regimens.

However, a number of experts noted that AIDS is seen as a much more “imminent” health threat than HPV/cervical cancer or other diseases to an individual, and that there would be greater willingness to be vaccinated. Further, already existing AIDS advocacy and awareness could drive very high demand for and uptake of an AIDS vaccine, and experts agreed that the availability of an AIDS vaccine is likely to further enhance such efforts.

Also, continued improvements in infrastructure to deliver HPV vaccines and other vaccines targeting adults and adolescents that may come online (such as tuberculosis vaccines) before an AIDS vaccine is licensed may facilitate uptake. The example of how pneumococcal vaccination programs achieved rapid scale-up by being added to existing pentavalent vaccination programs was cited and recent mass vaccination against meningococcal A meningitis which reached almost total coverage shows the potential for well-aligned policies, efforts and actors to ensure high coverage in front of a strongly perceived threat [^[[11]](#endnote-12)^]. Finally, continued improvements in overall health systems in LMICs could drive more effective immunization campaigns.

After discussions with experts and influenced by recent demand assessments for AIDS vaccines that used a similar approach [^[[12]](#endnote-13)^], coverage levels in line with a vaccination program reflective of current programs were proposed with tiered ages, starting with routine school-based vaccination of 10-year-olds at a relatively high level. As the model as currently built does not explore HIV/AIDS impact in persons younger than age 15, an aspect was built in to ensure that the proportion of persons vaccinated at age 10 “enter” the model protected by the vaccine at age 15.

Further, the difficulty in reaching consistent coverage in adults across age ranges, especially in those no longer reachable with school-aged programs, was considered through reduced coverage of older age tiers. The model does not allow differentiated age coverage, so a weighted average was used to approximate tiering in older populations.

The final tiered coverage assumptions for the base scenario reflects this updated approach, while maintaining an optimism reflective of strong advocacy and an enabling environment for AIDS vaccine implementation. Sensitivity analyses of coverage assumptions 10% and 20% above and below each sub-assumption provide context for increased or reduced vaccine coverage as well as for considerations around vaccine adherence.

**Target coverage: high-incidence populations in concentrated epidemics**

As reflected in the UNAIDS IFE, effective HIV/AIDS prevention programs for countries with epidemics heavily concentrated in populations with high incidences, including sex workers, men who have sex with men (MSM), and drug users, should be tailored to focus on those populations for maximum impact and efficiency. Similarly, AIDS vaccine implementation in such countries may also consider targeted programs to populations with high incidences. Such programs would be more cost-effective than widely vaccinating general populations of primarily lower incidence.

Experts noted that in concentrated epidemics, the nature of demand for an AIDS vaccine is likely to be different – those most likely to benefit will need to present themselves to health systems to be vaccinated, as opposed to receiving it through a school program. In that light, a number of experts recommended looking to coverage of existing AIDS prevention, treatment, and care interventions as a proxy for implementing an AIDS vaccine in concentrated epidemics. For example, vaccination could be linked to HIV testing programs. (This also included a discussion of whether a preventive vaccine could be given to somebody regardless of their HIV status; unless safety was tested in HIV-positive people, vaccination of high-incidence populations would be likely to require testing to ensure vaccination of naïve individuals only. However, given the lack of clarity around future clinical study designs and the lack of knowledge around safety and whether a vaccine candidate designed to be preventative might also provide therapeutic benefit to HIV positive individuals, this was not explored deeper.)

Some stakeholders pointed out the inconsistent accessibility of high-incident populations between different countries. While some stakeholders felt that enhanced awareness and health infrastructure should support the achievement of high coverage, others felt that stigma and in some cases even criminalization could stand in the way and that coverage and adherence assumptions should maintain a level of pessimism.

Current data on access to testing and other HIV prevention modalities for key populations presents a complicated picture. UNAIDS reports that median coverage of HIV services for gay men and other men who have sex with men (MSM) in the Asia/Pacific region stands at 58%; for European MSM that number is 50%. In 35 sub-Saharan African countries, an average of only 60% of female sex workers reported a recent HIV test in prior 12 months for which they learned their results [^[[13]](#endnote-14)^].

The base case of this work includes a 50% coverage rate in concentrated epidemics of at-risk populations for AIDS vaccines, with sensitivity analyses of 30, 40, 60, and 70% to provide context for increased or reduced vaccine coverage as well as for considerations around vaccine adherence.

**Rate at which target coverage is met (all epidemic types)**

Ideally, policies and infrastructures will be in place to ensure rapid access to AIDS vaccines very soon after licensure. A number of respondents noted that infrastructure to deliver HPV vaccines would be in place and could expedite AIDS vaccine delivery, given the similarity in likely target population (pre-adolescents, adolescents, and young adults). Further, high demand for an AIDS vaccine deriving from high levels of support and advocacy around HIV/AIDS in general could facilitate rapid access. At the same time, coverage goals used by Gavi: the Vaccine Alliance for newly introduced vaccines targeting children are in some cases less optimistic than the baseline coverage assumption or AIDS vaccines [^[[14]](#endnote-15)^].

Further, rapid scale-up of adult male circumcision in AIDS endemic countries has shown that an AIDS prevention tool that is relatively invasive and requires significant adherence can still reach high levels of coverage in a short period of time if political will is there to facilitate [^[[15]](#endnote-16)^]. More generally, progress in scaling up AIDS treatment in LMICs in the past five years has been remarkable, inspiring optimism for rolling out priority health interventions with strong political will [xiii].

An optimistic scale-up time of 6 years was incorporated into the Base case, with sensitivity analyses of 5 years (slightly more optimistic) and 10 years, from which to project the impact of increased or delayed scale-up.

*Costs (Table 2)*

The actual prices of recently introduced vaccines (HPV, rotavirus, pneumococcus) have varied significantly between the developed world and the developing world. Price per dose at introduction in developed countries can reach US $147 per dose (HPV vaccination, private sector, USA), reflecting the value in a high-income market [^[[16]](#endnote-17)^]. In developing countries, low costs have been driven by market-shaping through Gavi, purchase pools or co-financing such as advance market commitments, and by industry programs and commitments [^[[17]](#endnote-18)^, ^[[18]](#endnote-19)^, ^[[19]](#endnote-20)^,^[[20]](#endnote-21)^, ^[[21]](#endnote-22)^]. One expert noted that currently under Gavi, 20 low-income countries are provided vaccines gratis. Another noted that novelty, efficacy, and health benefit will play a role in cost, making the point that the cost for HPV vaccination are well-accepted due to its high efficacy and the breakthrough it represents. In the realm of HIV treatment, negotiated price reductions between the Clinton Health Access Initiative and drug manufacturers have significantly driven down purchase prices [^[[22]](#endnote-23)^].

Four price scenarios were created to reflect a broad spectrum of cost scenarios:

- **The base scenario** aligns most closely with the vaccine cost assumptions from the IFE model, with total cost per regimen of US $20 for LICs and a total cost per regimen of US $55 for MICs, considering a potential Gavi rollout in eligible countries that would likely result in tiered pricing by country income level.
- **The Higher price scenario** models a high cost per regimen reduction for LICs (US $35) and for MICs (US $100) and situates between the base scenario and the very high price scenario.
- **The very high price** (US $65 total cost per regimen for LICs, US $160 for MICs) represents possible challenges, including but not limited to expensive manufacturing, distribution and/or administration, or barriers to previously effective price-reducing strategies. This scenario also enables comparisons to a modeling exercise exploring the potential impact of an RV144-like vaccination program [^[[23]](#endnote-24)^] that had initially high efficacy with waning effect over time. The RV144 US $100 per dose assumption used in some of the models was not based on any tangible data on the RV144 regimen’s cost; it was simply a representative figure.
- **The Middle Income cost = Lower Income Cost scenario** of US $20 for all countries provides a “best case” by which costs are both low and equitable across all LICs and MICs regardless of epidemic type, supporting the potential for inexpensive manufacturing, distribution, administration of successful mechanisms, and advocacy to ensure affordability.

**Two- and four-dose vaccine regimens** building on the base-case scenario adjust the total cost accordingly as well as the implementation cost to reflect the higher or lower cost based on number of doses.

Experts recommended looking to HPV vaccine implementation costs as references for the implementation of AIDS vaccines [^[[24]](#endnote-25)^]. At the same time, noting that HPV vaccine demonstrations are often building new program infrastructure, an expert in vaccine delivery pointed out that implementation costs for new vaccines can be quite high when new systems are necessary (US $3–4 per dose), while that cost can be heavily reduced when an existing system can be utilized (US $0.10–0.30 per dose).

Reductions over time in implementation cost reflect increasing efficiencies in vaccine delivery after initial startup costs and increased synergies with other vaccinations or health programs. Rate of cost reduction was influenced by those of other vaccines and TB diagnostics, which saw 40% reductions in just a few years [^[[25]](#endnote-26)^].

**Table S3: Vaccine Cost Scenarios**

|  | Doses | Low-Income Countries (LICs) | | | Middle-Income Countries (MICs) | | |
| --- | --- | --- | --- | --- | --- | --- | --- |
|  |  | **Per Dose*** | **Implementation*** | **Total for Regimen*** | **Per Dose*** | **Implementation*** | **Total*** |
| Launch scenarios | | | | | | | |
| Base^1^ | 3 | 5 | 5 | 20 | 15 | 10 | 55 |
| Higher price^2^ | 3 | 10 | 5 | 35 | 30 | 10 | 100 |
| Very high price^3^ | 3 | 20 | 5 | 65 | 50 | 10 | 160 |
| Middle income price = lower income price^4^ | 3 | 5 | 5 | 20 | 5 | 5 | 20 |
| 2-dose regimen | 2 | 5 | 4 | 14 | 15 | 8 | 38 |
| 4-dose regimen | 4 | 5 | 6 | 26 | 15 | 12 | 72 |
| 10-years-out scenarios | | | | | | | |
| Base | 3 | 3 | 3 | 12 | 10 | 7 | 37 |
| Higher price | 3 | 5 | 3 | 18 | 15 | 7 | 52 |
| Very high price | 3 | 10 | 3 | 33 | 30 | 7 | 97 |
| Middle income price = lower income price | 3 | 3 | 3 | 12 | 3 | 3 | 12 |
| 2-dose regimen | 2 | 3 | 2 | 8 | 10 | 5 | 25 |
| 4-dose regimen | 4 | 3 | 4 | 16 | 10 | 9 | 49 |

**Impact on AIDs-related deaths: Three Scenarios**

In the *Current Trends* scenario, incremental scale-up of existing HIV/AIDS interventions results in a continuing reduction in AIDS-related deaths due to the success of treatment and care programs to date, followed by a rise and then flat trajectory in deaths as programs reach coverage targets as a percentage of HIV positive individuals but the absolute number of HIV positive individuals on treatment is reduced. Without a vaccine the annual number of deaths drops below 771,000 in 2020 but then rises slightly over time, reaching just over 1 million in 2070. (Figure S1) AIDS deaths in the 50% Scale-Up of IFE scenario follow a similar trajectory as Current trends before diverging around 2030, at which point new infections begin a downward trajectory that extends to 2070. In the IFE scenario, the initial drop in annual AIDS-related deaths (to around 650,000) is more pronounced, with the subsequent rise to nearly 769,00 before a sharp drop in AIDS-related deaths after an initially large drop below 445,000 by 2070. Including an AIDS vaccine (base-case characteristics, see Table A2) would reduce the annual number of new HIV infections in 2070 by 61% to around 396,000, by 61% to around 289,000, by 56% to around 194,000 when implemented with Current Trends, Full Scale-up of IFE, and 50% Scale-up of IFE, respectively. Overall, an AIDS vaccine would reduce the number of cumulative AIDS deaths from 2027 to 2070 by more than 9 million, 8 million, and 5 million if applied to Current Trends, Full Scale-up of IFE, and 50% Scale-up of IFE, respectively (Figure S1).


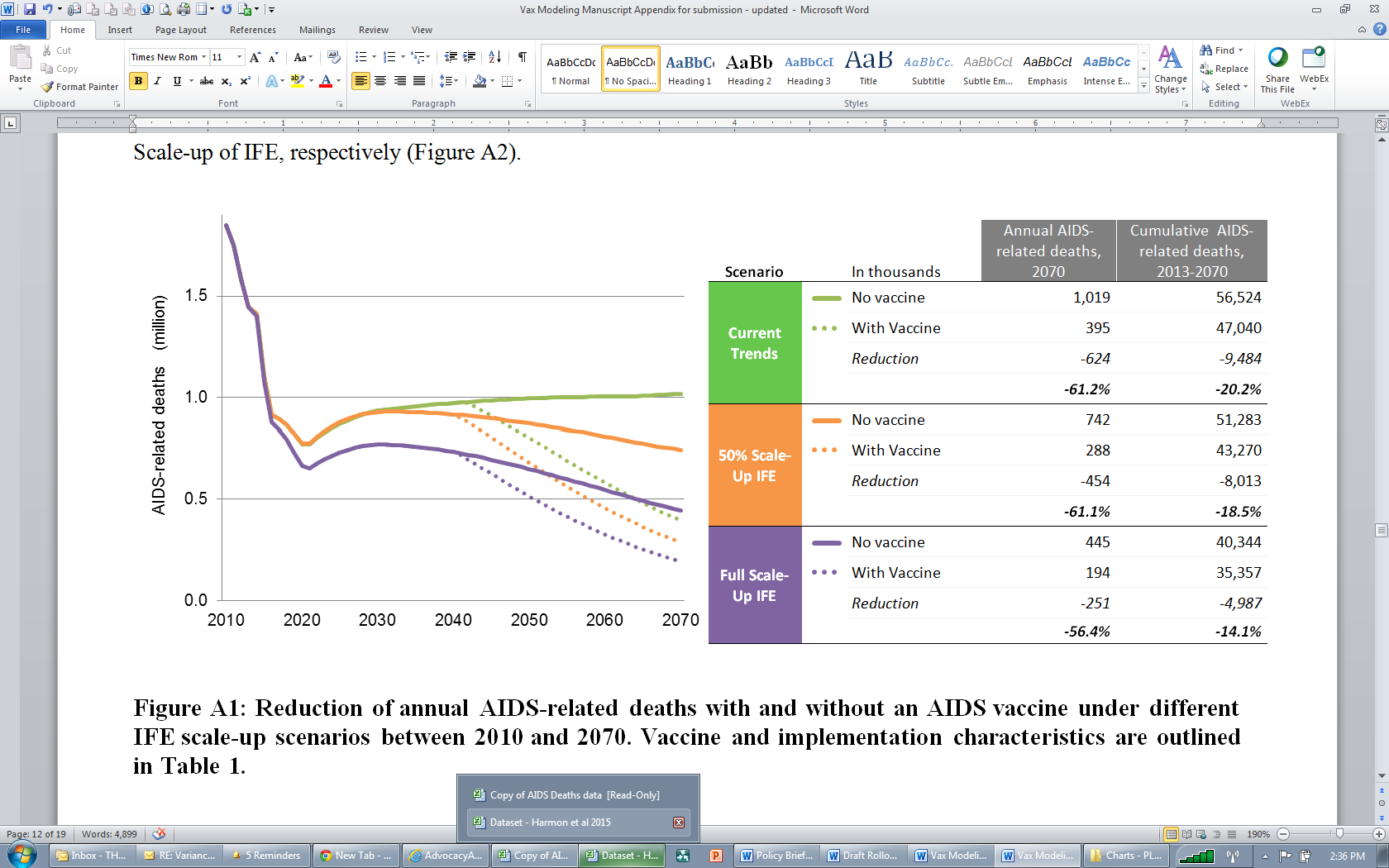


**Figure S1: Reduction of annual AIDS-related deaths with and without an AIDS vaccine under different IFE scale-up scenarios between 2010 and 2070. Vaccine and implementation characteristics are outlined in Table 1.**

**Cost-effectiveness – Additional Analyses**

*Cost-effectiveness related to vaccine efficacy*

Our model disaggregated cost-effectiveness analysis between LICs and MICs. Cost-effectiveness analyses in the primary article focus on LICs. In LICs, differing costs, vaccine efficacy, and dose considerations presented varying levels of cost-effectiveness, providing a nuanced picture of the interplay between vaccine characteristics and cost-effectiveness. In MICs almost all vaccine scenarios showed cost-effectiveness under WHO thresholds.

Under base vaccine characteristics, a vaccine is both cost-effective (≤3x gross national income (GNI) per capita or US $28,404) and highly cost-effective (≤1 GNI per capita or US $9,468) under all cost scenarios in MICs. (Figure S2)


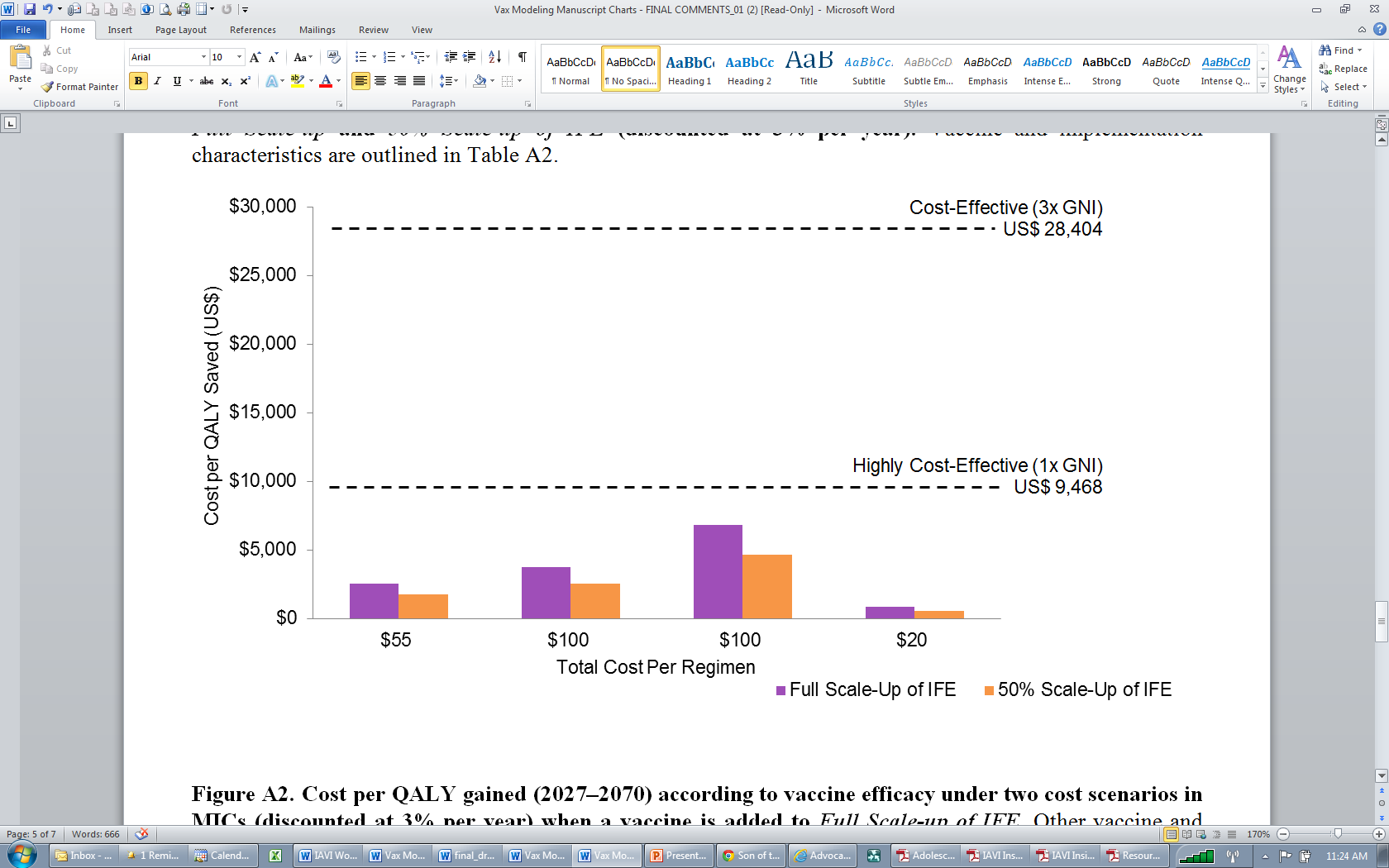


Figure S2. Cost per QALY gained with an AIDS vaccine (base-case characteristics) in MICs added to *Full Scale-up* and *50% Scale-up of IFE* (discounted at 3% per year). Vaccine and implementation characteristics are outlined in Table S2.

Focusing on MICs, Figure S3 illustrates that, at baseline cost (US $55 per regimen), an AIDS vaccine would be highly cost-effective in MICs under all efficacy sensitivity scenarios (30–90%). Under the higher price scenario (US $160 per regimen), an AIDS vaccine would be cost-effective under all efficacy scenarios, but would need to be around 60% efficacious to be highly cost-effective in MICs.


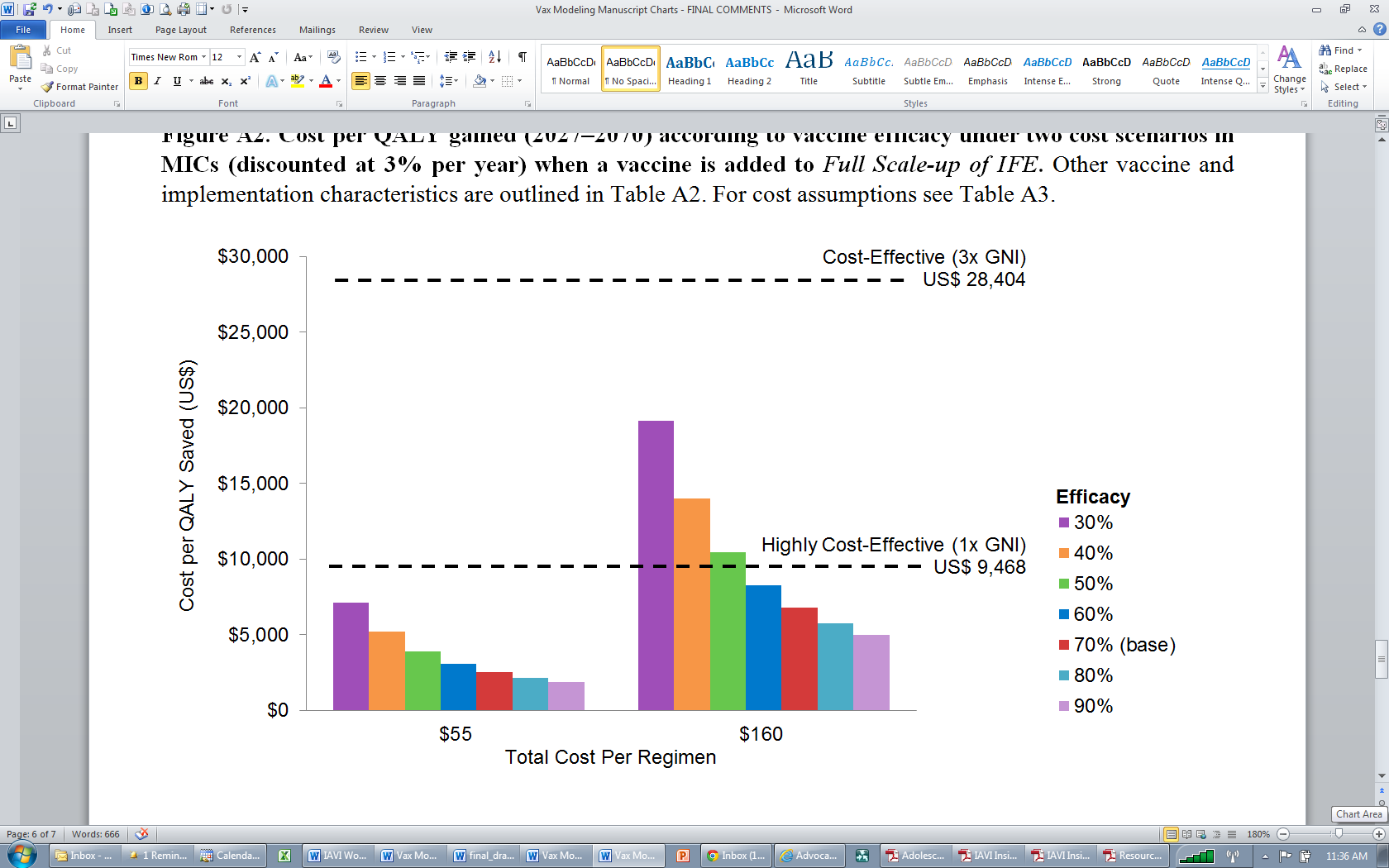


Figure S3. Cost per QALY gained (2027–2070) according to vaccine efficacy under two cost scenarios in MICs (discounted at 3% per year) when a vaccine is added to *Full Scale-up of IFE*. Other vaccine and implementation characteristics are outlined in Table S2. For cost assumptions see Table S3.

*Cost-effectiveness related to duration of protection in LICs and MICs*

Figures S4 and S5 show the relation between cost-effectiveness and duration of protection of an AIDS vaccine in LICs and MICs. In LICs, a vaccine of base characteristics is highly cost-effective under all but a low vaccine duration of 3 years, and is cost effective under all durations and prices except in a very low duration and very high price scenario. In MICs, a vaccine of base characteristics is highly cost-effective under almost all scenarios, with only a vaccine of very high price and only 3 years duration not achieving the highly cost-effective threshold.


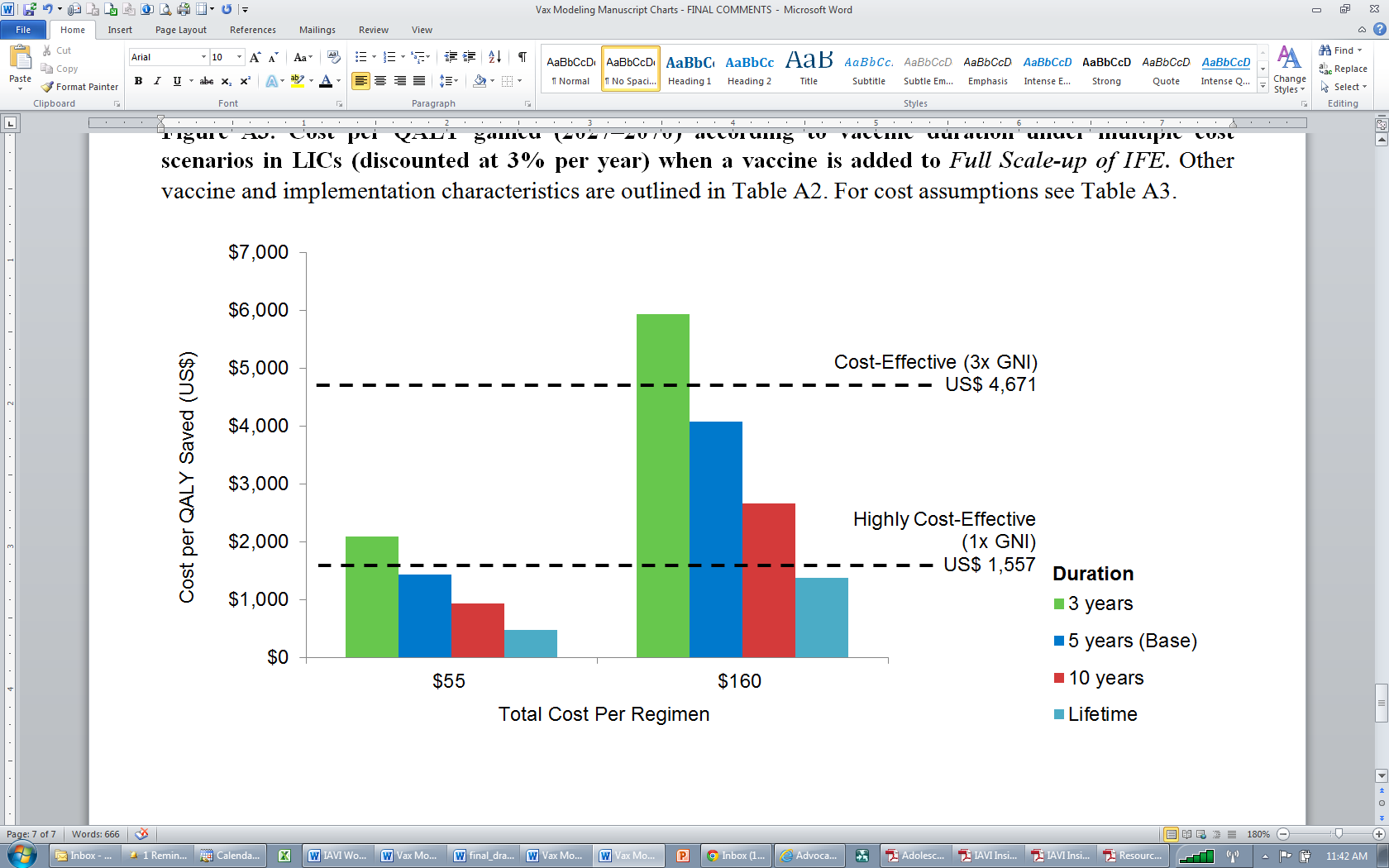


**Figure S4. Cost per QALY gained (2027–2070) according to vaccine duration under multiple cost scenarios in LICs (discounted at 3% per year) when a vaccine is added to *Full Scale-up of IFE*.** Other vaccine and implementation characteristics are outlined in Table S2. For cost assumptions see Table S3.


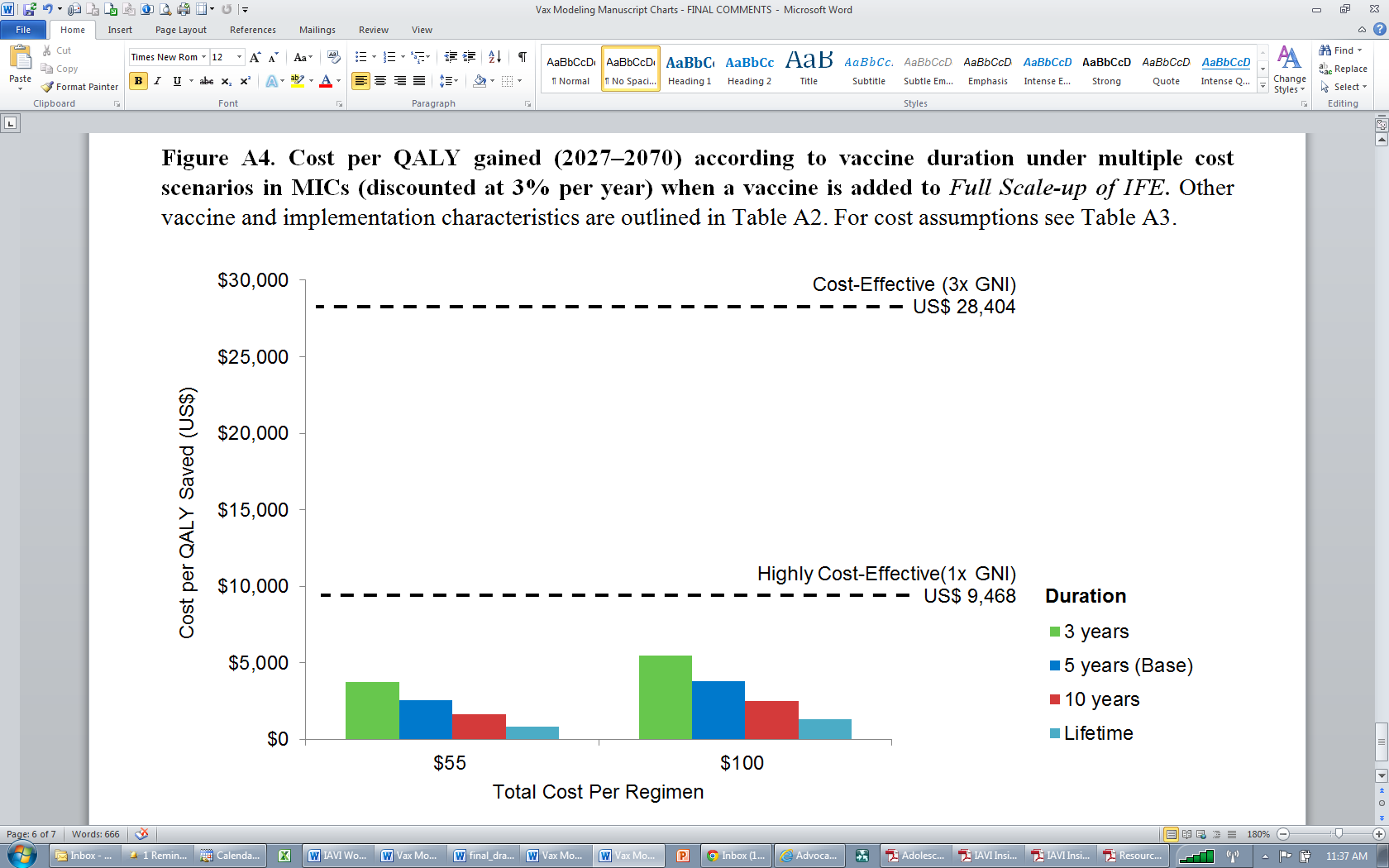


Figure S5. Cost per QALY gained (2027–2070) according to vaccine duration under multiple cost scenarios in MICs (discounted at 3% per year) when a vaccine is added to *Full Scale-up of IFE*. Other vaccine and implementation characteristics are outlined in Table S2. For cost assumptions see Table S3.

**Experts consulted to inform assumptions**

Aurum Institute

Gavin Churchyard, Chief Executive Officer, interviewed 5/2/14

Bill and Melinda Gates Foundation

Geoff Garnett, Deputy Director, Data and Metrics, Integrated Delivery, interviewed 5/1/14

Gavi: the Vaccine Alliance

Lauren Fransel, Senior Specialist, Policy & Market Shaping, interviewed 5/27/14

Santiago Cornejo, Senior Specialist, Immunization Financing, interviewed 5/27/14

Global Fund to Fight AIDS, Tuberculosis and Malaria

[Nicolas Bidault](mailto:Nicolas.bidault@theglobalfund.org), Senior Manager, Strategic Information, interviewed 6/18/14

Mehran Hosseini, Senior Specialist Disease & Impact Modeling, interviewed 6/18/14

Kenya AIDS Vaccine Initiative

Omu Anzala, Programme Director, interviewed 5/12/14

Uganda Virus Research Institute

Pontiano Kaleebu, Director, interviewed 5/12/14

University of the Witwatersrand, Wits Reproductive Health and HIV Institute

Helen Rees, founder and Executive Director, interviewed 7/11/14

Representatives of pharmaceutical vaccine manufacturers

Names withheld

References

1. . Stover J, Hallett TB, Wu Z, Warren M, Gopalappa C, Pretorius C, et al. How can we get close to zero? The potential contribution of biomedical prevention and the investment framework towards an effective response to HIV. PLoS One. .L2014;9(11):e111956. [↑](#endnote-ref-2)
2. . US Military HIV Research Program. RV144 Trail. Available at: <http://www.hivresearch.org/research.php?ServiceID=13>. Last accessed on 25 June 2015. [↑](#endnote-ref-3)
3. . Newman PA, Logie C. HIV vaccine acceptability: a systematic review and meta-analysis. AIDS. 2010;24:1749–56. [↑](#endnote-ref-4)
4. . Hankins CA, Glasser JW, Chen RT. Modeling the impact of RV144-like vaccines on HIV transmission. Vaccine. 2011;29(36):6069–71. [↑](#endnote-ref-5)
5. . AVAC: Global Advocacy for HIV Prevention. AIDS vaccine science for busy advocates – current AIDS vaccine R&D pipeline. Available at: <http://www.avac.org/infographic/aids-vaccine-science-busy-advocates-current-aids-vaccine-rd-pipeline>. Last accessed on 25 June 2015. [↑](#endnote-ref-6)
6. . National Institute of Allergy and Infectious Disease. Bulletin: In South Africa, RV144 vaccine regimen induces immune responses similar to those seen in Thailand. Available at: <http://www.niaid.nih.gov/news/newsreleases/2014/Pages/RV144HIVvax.aspx>. Last accessed on 25 June 2015. [↑](#endnote-ref-7)
7. . Ladner J, Besson M-H, Rodrigues M, Audureau E, Saba J. Performance of 21 HPV vaccination programs implemented in low- and middle-income countries, 2009–2013. BMC Public Health. 2014;14:670. [↑](#endnote-ref-8)
8. . http://www.biomedcentral.com/1471-2458/14/670 [↑](#endnote-ref-9)
9. . Centers for Disease Control and Prevention. Flu vaccination coverage, United States, 2013–14 influenza season. Available at: <http://www.cdc.gov/flu/fluvaxview/coverage-1314estimates.htm>. Last accessed on 25 June 2015. [↑](#endnote-ref-10)
10. . Gavi: The Vaccine Alliance. Gavi Goal level indicators. Available at [www.gavi.org/results/gavi_alliance_goal_level_indicators/](http://www.gavi.org/results/gavi_alliance_goal_level_indicators/). Last accessed on 29 June 2015. [↑](#endnote-ref-11)
11. . Kristiansen PA, Diomande F, Ba FE, Sanou I, Ouedraogo AS, Ouedraogo R, et al. Impact of the serogroup A meningococcal conjugate vaccine, MenAfriVac, on carriage and herd immunity. Clin Infect Dis. 2013;56(3):354–63. [↑](#endnote-ref-12)
12. . Marzetta CA, Wrobel SJ, Singh KK, Russell ND, Esparza J. P15-09. The potential global market size and public health value of hypothetical HIV-1 preventive vaccines with different levels of efficacy. Retrovirology. 2009;6(suppl3):P210. [↑](#endnote-ref-13)
13. . UNAIDS. Gap report. Available at: <http://www.unaids.org/en/resources/campaigns/2014/2014gapreport/gapreport>. Last accessed on 25 June 2015. [↑](#endnote-ref-14)
14. . Gavi: The Vaccine Alliance. Vaccine goal indicators. Available at: <http://www.gavi.org/results/goal-level-indicators/vaccine-goal-indicators/>. Last accessed on 25 June 2015. [↑](#endnote-ref-15)
15. . World Health Organization. WHO Progress Brief – Voluntary medical male circumcision for HIV prevention in priority countries in East and Southern Africa. Available at: <http://www.who.int/hiv/topics/malecircumcision/male-circumcision-info-2014/en/>. Last accessed on 25 June 2015. [↑](#endnote-ref-16)
16. . The Henry J. Kaiser Family Foundation. The HPV vaccine: access and use in the U.S. Available at: <http://kff.org/womens-health-policy/fact-sheet/the-hpv-vaccine-access-and-use-in/>. Last accessed on 25 June 2015. [↑](#endnote-ref-17)
17. . Gavi: The Vaccine Alliance. Market-shaping goal indicators. Available at: <http://www.gavi.org/results/goal-level-indicators/market-shaping-goal-indicators/>. Last accessed on 25 June 2015. [↑](#endnote-ref-18)
18. . The World Health Organization. Brief 12: The vaccine market – pooled procurement. Available at: <http://www.who.int/immunization/programmes_systems/financing/analyses/Brief_12_Pooled_Procurement.pdf>. Last accessed on 25 June 2015. [↑](#endnote-ref-19)
19. . Gavi: The Vaccine Alliance. Pneumococcal AMC. Available at: <http://www.gavi.org/funding/pneumococcal-amc/>. Last accessed on 25 June 2015. [↑](#endnote-ref-20)
20. . The Gardasil Access Program. Information to applicants about the GARDASIL^®^ Access Program. Available at: <http://www.gardasilaccessprogram.org/x/File/200902%20GARDASIL%20Access%20Program-%20Information%20to%20applicants.pdf>. Last accessed on 25 June 2015. [↑](#endnote-ref-21)
21. . Bhan A, Green SK. Balancing safety, efficacy and cost: Improving rotavirus vaccine adoption in low- and middle-income countries. J Glob Health. 2011;1(2):148–53. [↑](#endnote-ref-22)
22. . Tagar E, Sundaram M, Condliffe K, Matatiyo B, Chimbwandira F, Chilima B et al. Multi-country analysis of treatment costs for HIV/AIDS (MATCH): Facility-level ART unit cost analysis in Ethiopia, Malawi, Rwanda, South Africa and Zambia. PLoS One. 2014;9(11):e108304. [↑](#endnote-ref-23)
23. . Chen RT, Glasser JW, Hankins C. AIDS Vaccine 2010 Satellite Symposium. 2011;29(36):6069–143. [↑](#endnote-ref-24)
24. . Levin A, Wang SA, Levin C, Tsu V, Hutubessy R. Costs of introducing and delivering HPV vaccines in low and lower middle income countries: Inputs on GAVI policy and introduction grant support to countries. PLoS One. 2014;9(6):e101114. [↑](#endnote-ref-25)
25. . Treatment Action Group. GeneXpert Rapid TB test price reduced in historic agreement. Available at: <http://www.treatmentactiongroup.org/tagline/2012/fall/genexpert-rapid-tb-test-price-reduced-historic-agreement>. Last accessed on 25 June 2015. [↑](#endnote-ref-26)
